# Recent Advances in Tumor Microenvironment Hydrogen Peroxide-Responsive Materials for Cancer Photodynamic Therapy

**DOI:** 10.1007/s40820-019-0347-0

**Published:** 2020-01-03

**Authors:** Nan Yang, Wanyue Xiao, Xuejiao Song, Wenjun Wang, Xiaochen Dong

**Affiliations:** 1 Key Laboratory of Flexible Electronics (KLOFE) and Institute of Advanced Materials (IAM), School of Physical and Mathematical Sciences, Nanjing Tech University (Nanjing Tech), Nanjing, 211800 People’s Republic of China; 2 School of Physical Science and Information Technology, Liaocheng University, Liaocheng, 252059 People’s Republic of China; 3 School of Chemistry and Materials Science, Nanjing University of Information Science and Technology, Nanjing, 210044 People’s Republic of China

**Keywords:** Tumor microenvironment, H_2_O_2_-responsive, Cancer, Nanomaterials, Photodynamic therapy

## Abstract

Photodynamic therapy (PDT), as one of the noninvasive clinical cancer phototherapies, suffers from the key drawback associated with hypoxia at the tumor microenvironment (TME), which plays an important role in protecting tumor cells from damage caused by common treatments. High concentration of hydrogen peroxide (H_2_O_2_), one of the hallmarks of TME, has been recognized as a double-edged sword, posing both challenges, and opportunities for cancer therapy. The promising perspectives, strategies, and approaches for enhanced tumor therapies, including PDT, have been developed based on the fast advances in H_2_O_2_-enabled theranostic nanomedicine. In this review, we outline the latest advances in H_2_O_2_-responsive materials, including organic and inorganic materials for enhanced PDT. Finally, the challenges and opportunities for further research on H_2_O_2_-responsive anticancer agents are envisioned.

## Highlights

- The reaction mechanism of various kinds of nanomaterials with endogenous H_2_O_2_ is outlined.
- The design and application guideline for various H_2_O_2_-responsive nanomaterials in photodynamic therapy (PDT) are reviewed.
- The development and prospect of various H_2_O_2_-response nanomaterials for PDT and clinical application are envisioned.

## Graphical abstract

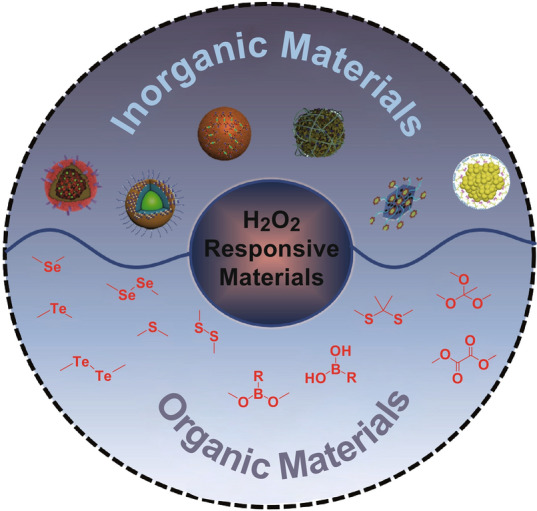

## Introduction

Cancer is one of the leading threats to human health and development [1]. Traditional cancer therapies, including surgery, chemotherapy, and radiotherapy, have their inherent drawbacks despite being used clinically for decades [2]. For example, surgery is generally applied to remove tumors for biopsy. But it is not applicable for leukemia and metastatic cancers. Chemotherapy and radiotherapy are usually the main treatments for terminal cancer. However, these traditional therapeutic methods are always accompanied by serious drug resistance and severe side effects, which cause the patients suffering [3]. With the development of nanomaterials, photodynamic therapy (PDT), employing a light-excited photosensitizer (PS) to generate reactive oxygen species (ROS) in the presence of oxygen (O_2_), has received increasing attention owing to its low systemic toxicity, high selectivity, and minimal invasiveness compared with traditional therapies [4, 5].

TME is mainly composed of fibroblasts and myofibroblasts, neuroendocrine cells, adipose cells, immune, and inflammatory cells, the blood and lymphatic vascular networks, and extracellular matrix (ECM) [6]. Changes in the physiological state and function of TME lead to tumor progression. When TME is in its initial normal state, further invasion and metastasis of the tumor cells will be prevented by TME, but once TME is destroyed into an irreversible situation, it will become an accomplice to cancer deterioration. In addition, TME has been identified to be the indicator for determining abnormal tissue function and plays a key role in the subsequent evolution of persistent and advanced malignancies [7]. Due to the interaction between different stromal cells and active factors blocking TME, traditional strategies for tumor intervention and treatment are often unsatisfied. The blocking mechanism induced by the treatments could be quickly adapted and balanced by TME, which results in continued development and deterioration of cancer cells. Different from the traditional treatment strategies, PDT can promote cancer cell death by increasing the concentration of ROS in TME.

Singlet oxygen (^1^O_2_) is one of the most important ROS catalyzed by O_2_ in PDT [8]. However, TME is always hypoxic due to the characteristics of cancer cells including unlimited multiplication, evasion from growth suppressors, resisting apoptosis, stimulating angiogenesis, and elimination of cell energy limitation [9]. During the cell proliferation process, a large amount of O_2_ is consumed. Although the number of blood vessels is increased, the partial pressure of O_2_ in the blood vessels is greatly reduced and the O_2_ replenishing ability is lowered, which finally limits the efficiency of PDT [10, 11]. To date, various methods have been developed to overcome tumor hypoxia and improve the efficiency of tumor treatment, such as hyperbaric O_2_ therapy, O_2_ delivery based on perfluorohexane, O_2_ generated by catalase-like nanomaterials, and anoxic therapy [12]. Furthermore, various intelligent systems that can respond to external stimuli (such as heat, irradiation or microwave) and internal stimuli (TME factors such as H_2_O_2_ or certain enzymes) are developed to improve the tumor-targeting efficiency of PSs, reduce the side effects, and enhance the efficacy of PDT [13].

H_2_O_2_ (half-life about 1 ms) is relative stable as compared with other ROS (half-life < 1 μs). In addition, H_2_O_2_ acts as extracellular and intracellular signaling molecule that mediates multiple effects in biological systems, including recruitment of immune cells to damaged areas and cell migration. H_2_O_2_ is obtained from mitochondria generated superoxide ions in a process that is catalyzed by the overexpressed superoxide dismutase (SOD). Compared with normal cells, cancer cells show increased generation rate of H_2_O_2_ (up to 0.5 nmol/10^4^ cells/h) [8], resulting in a higher level of H_2_O_2_ in the tumor than normal tissues. In addition to its essential role in cellular signaling, overproduced H_2_O_2_ has been exploited as a major precursor for highly active ROS, such as hydroxyl radical, peroxynitrite, and hydrochlorides. Importantly, researchers have found that H_2_O_2_ accumulated in tumors can be decomposed to generate O_2_ under certain conditions, which is used to supplement the O_2_ required for PDT treatment. Since the accumulation of H_2_O_2_ can increase the oxidative stress and reflect the development of many diseases, taking H_2_O_2_ as a cancer diagnostic marker as well as a therapeutic target presents tremendous theranostic potential.

**Scheme 1:**
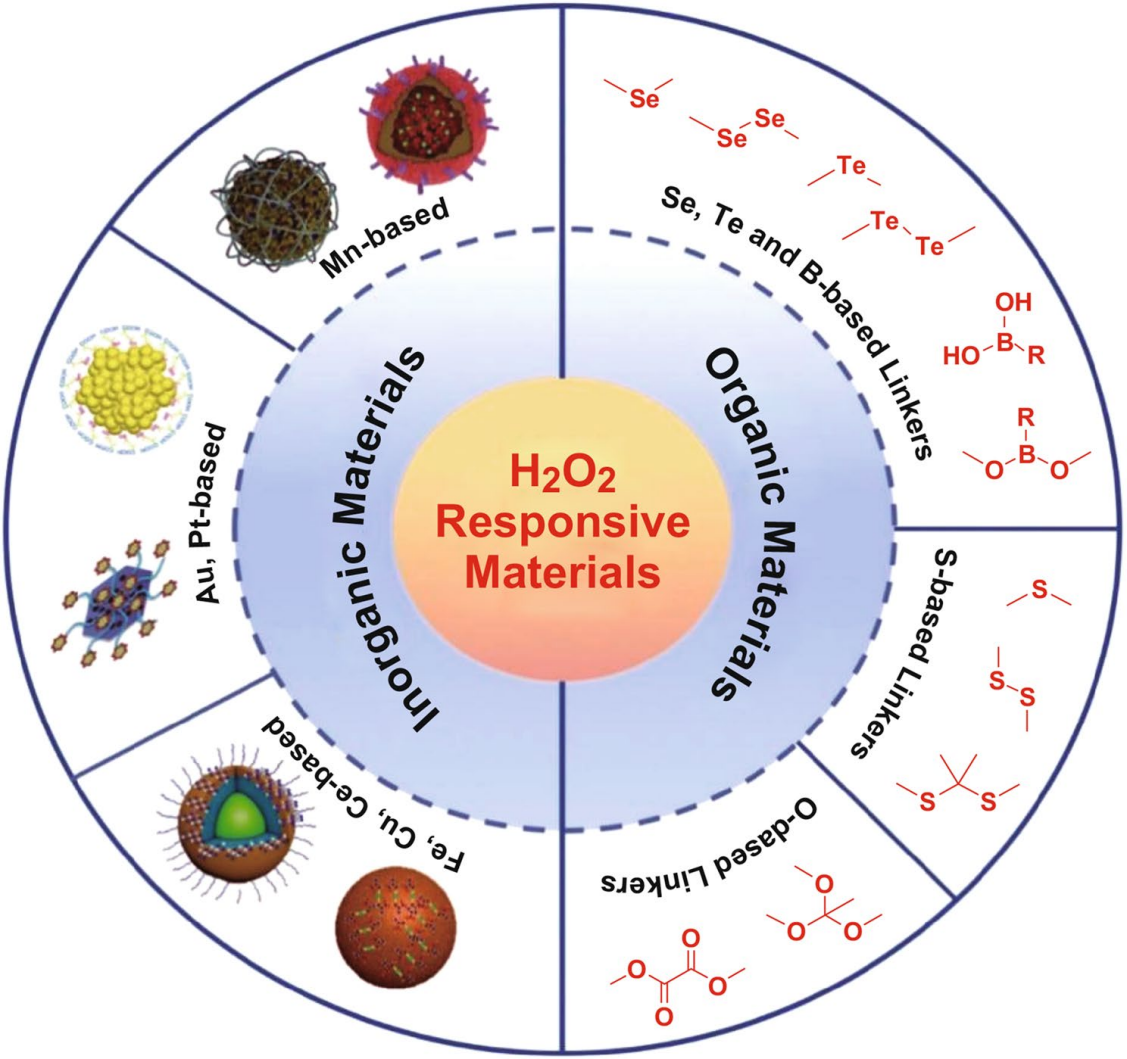
Summary of H_2_O_2_-responsive materials for enhanced photodynamic therapy

Up to now, many H_2_O_2_-responsive nanoplatforms based on inorganic or organic materials have been developed for enhancing anticancer PDT [14, 15]. Due to the unique physicochemical properties, easy surface functionalization and good biocompatibility, inorganic materials have been widely utilized as therapeutic agents in biomedical field [16]. Simultaneously, phthalocyanine, boron dipyrromethene (BODIPY), porphyrin, chlorin e6 (Ce6), and their derivatives are the most common organic photoactive PSs in PDT due to their low dark toxicity, high stability, intense adsorption band, and high absorbance coefficient [17]. Here, we describe different categories of H_2_O_2_-responsive phototherapeutic platforms for cancer treatments (Scheme 1).

## Inorganic Materials for H_2_O_2_-Responsive Photodynamic Therapy

Hypoxia at the tumor site has been reported to be the main cause of limited PDT efficiency. Strategies such as delivering O_2_ to the tumor site via perfluorocarbon-based O_2_ carriers or reducing O_2_ consumption have been developed to relieve tumor hypoxia, thus improving O_2_ involved therapies. Meanwhile, taking advantage of high concentration of endogenous H_2_O_2_, in situ production of O_2_ inside the tumor by catalyst could be a more effective approach to overcome tumor hypoxia and enhance PDT. Inorganic nanomaterials have attracted increasing attention owing to their unique physical/chemical properties, versatile synthetic strategies, and easy surface functionalization [18]. Inorganic therapeutic agents based on manganese (Mn), gold (Au), platinum (Pt), iron (Fe), copper (Cu), cerium (Ce), chromium (Cr), bismuth (Bi), vanadium (V), titanium (Ti), cobalt (Co), and lanthanides have been applied to external or internal stimuli-responsive cancer therapies. Besides, mesoporous silica (Si)-based materials, carbon-based materials like carbon dots (CDs) and semiconductors materials such as quantum dots (QDs) have also been utilized for cancer therapy [19]. In this section, endogenous H_2_O_2_-responsive inorganic materials for enhanced PDT, including Mn-, Au-, Pt-, Fe-, Cu-, and Ce-based materials, will be summarized.

### Mn-Based Materials

**Fig. 1:**
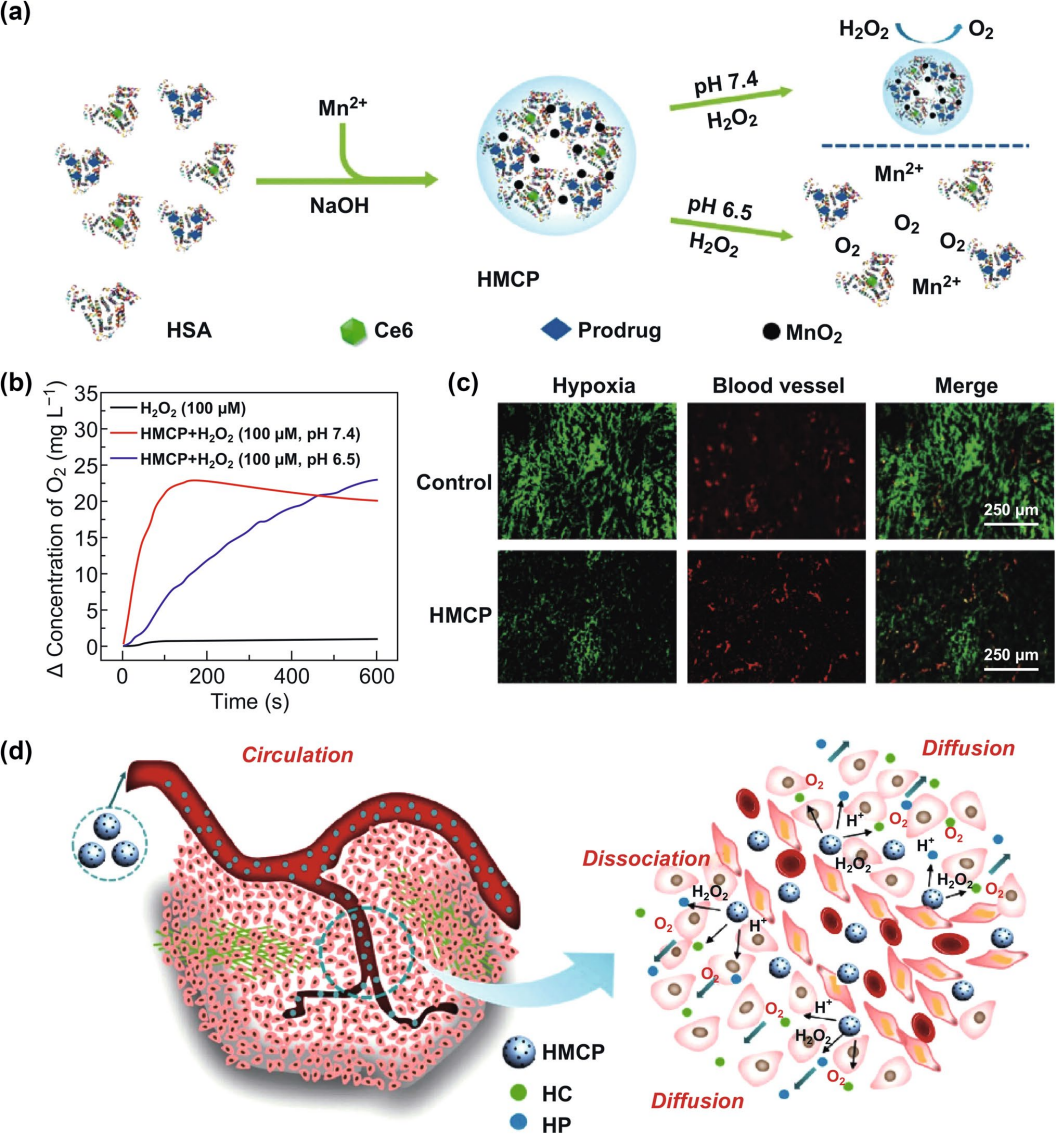
**a** Schematic representation of the formation of HMCP nanoparticles. **b** HMCP was added at different pH values to generate oxygen in H_2_O_2_ solution (100 × 10^−6^ M). The decrease in oxygen concentration in the sample at pH 7.4 (after about 150 s) is due to the rapid consumption of H_2_O_2_. **c** Representative immunofluorescence images of tumor sections after hypoxia staining. Hypoxic areas and blood vessels were stained with anti-pipemole antibody (green) and anti-CD31 antibody (red). **d** Scheme of the disintegration of HMCP nanoparticles by in situ chemical reaction in the TME for synergistic PDT and chemotherapy. Adapted with permission from Ref. [14]. Copyright 2016 Wiley–VCH

In recent years, Mn-based nanostructures have attracted considerable interests in bio-applications. Manganese dioxide (MnO_2_), the most common structure of manganese ions, shows high reactivity to acid and glutathione (GSH), being utilized as pH- or GSH-responsive drug delivery systems. Meanwhile, MnO_2_ nanoconstructs could decompose tumor endogenous H_2_O_2_ into O_2_, relieving tumor hypoxia. Different kinds of MnO_2_ nanoconstructs have been reported and made great progress in TME-enhanced tumor therapies [20]. Based on this, a unique multifunctional nanoplatform responsive to multiple parameters of TME with improved PDT efficiency has been reported. Multifunctional pH-/H_2_O_2_-responsive nanoparticles (NPs) named HSA-MnO_2_-Ce6&Pt (HMCP) with an average size of about 50 nm were synthesized via a simple one-step biomineralization method (Fig. 1a) [14]. These HMCPs were able to decompose endogenous H_2_O_2_ to generate O_2_ in situ for overcoming tumor hypoxia-associated resistance of PDT **(**Fig. 1b, c). On the other hand, HMCP could be dissociated into individual albumin-based complexes with smaller sizes (< 10 nm) in the acidic TME, resulting in enhanced intratumoral permeability for improved therapeutic outcomes of the synergistic PDT/chemotherapy **(**Fig. 1d). This nano-treatment platform is designed with MnO_2_ as the core of nanomaterials.

**Fig. 2:**
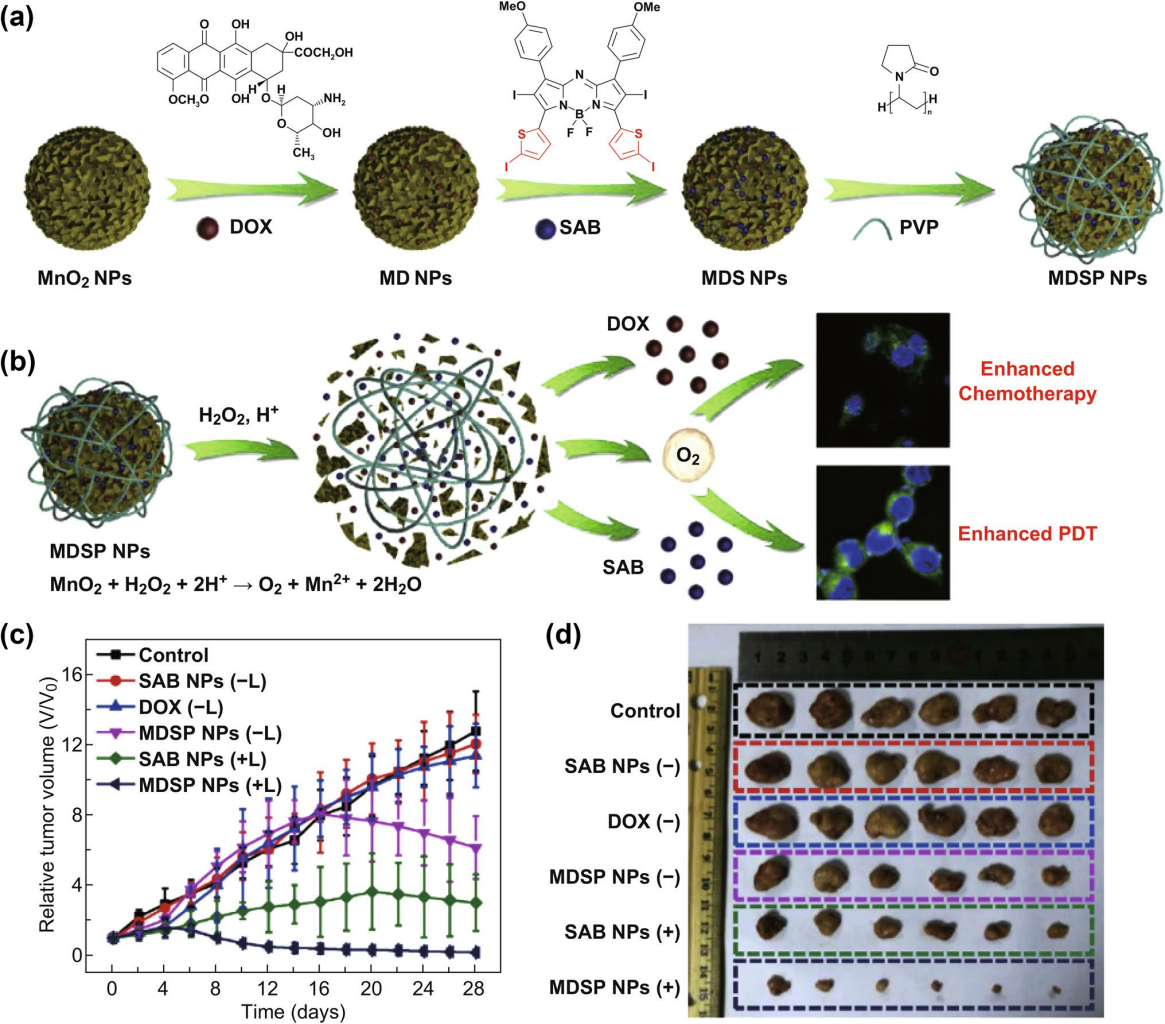
**a** Schematic diagram of MDSP NPs manufacturing. **b** Schematic illustration of MDSP NPs for TME-responsive chemo/photodynamic therapy. **c** Tumor growth curves of differently treated mice (6 mice/group). **d** Tumor photographs collected from mice in different treatment groups. Adapted with permission from Ref. [21]. Copyright 2019 Elsevier Ltd

Another strategy for designing nano-multi-functional platforms is to use MnO_2_ as nanoshell. Our group specifically designed a H_2_O_2_-responsive degradable nanoplatform by co-loading PS aza-BODIPY (SAB) and anticancer drug doxorubicin (DOX) into the hydrangea-structured MnO_2_ NPs for chemo/photodynamic/photothermal synergistic therapy (Fig. 2a). MnO_2_ in MDSP NPs can react with H_2_O_2_ and H^+^ in TME to generate oxygen and overcome tumor hypoxia (Fig. 2b) [21]. Injection of MDSP NPs combined with laser radiation showed the highest tumor inhibition efficiency. Both in vitro and in vivo studies demonstrated the promotion of TME-responsive oxygen-self-generation and excellent chemo/photodynamic/photothermal synergistic therapy of MDSP NPs (Fig. 2c–d).

**Fig. 3:**
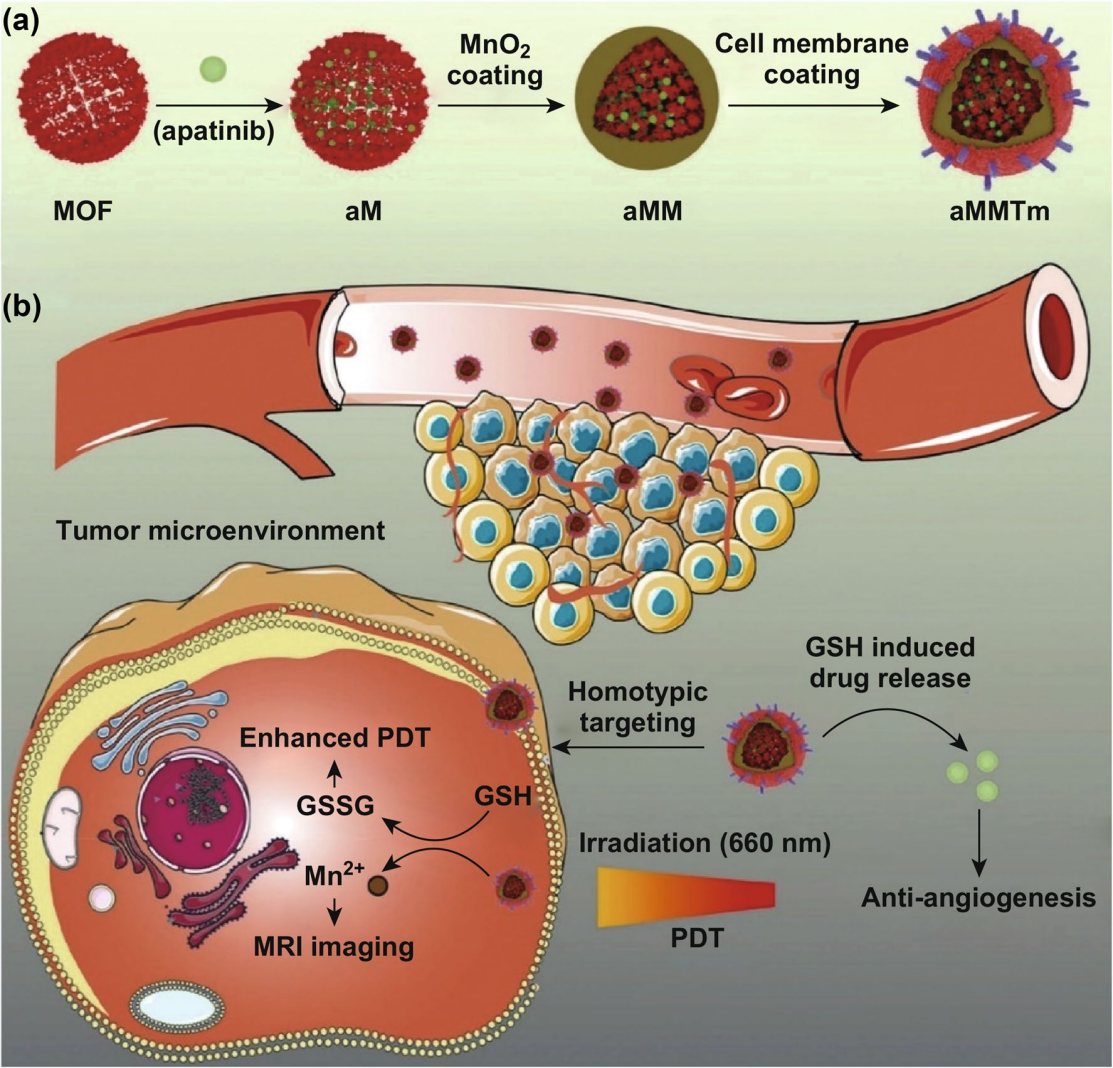
**a** Porphyrin metal–organic framework nanoparticle aMMTm was constructed by drug loading, MnO_2_ coating and tumor cell membrane decoration. **b** The proposed mechanism of action of aMMTm in mouse tumor model. Tumor cell membrane camouflage can help the early removal of nanoparticles and target tumor through homotypic affinity. At tumor site, MnO_2_ shell reacts with GSH in the microenvironment or tumor cells, consuming excess GSH and enhancing the action of PDT. Simultaneously produced Mn^2+^ can be used as MRI contrast agent. When MnO_2_ shell degrades, the released apatinib neutralizes PDT-induced revascularization and prevents tumor progression. Adapted with permission from Ref. [22]. Copyright 2019 WILEY–VCH

In addition to tumor hypoxia, PDT also suffers from neutralization by excess GSH in the TME. Reducing tumor GSH levels could benefit to enhance PDT efficiency. It was found that MnO_2_ could consume the intratumoral GSH. For example, Nie’s group developed a photosensitive porphyrinic Zr-MOF (PCN-224) nanostructure, which was loaded with vascular endothelial growth factor receptor 2 (VEGFR2) inhibitor apatinib and coated with a MnO_2_ layer (aMMTm, Fig. 3a) [22]. The tumor-targeting aMMTm NPs could be utilized as drug delivery vehicle and the MnO_2_ layer could neutralize excessive intratumoral GSH. Both in vitro and in vivo experiment showed the excellent anticancer efficiency of aMMTm NPs when compared with NPs without targeting ligand or MnO_2_ (Fig. 3b). Most importantly, Mn-based nanomaterials not only specifically trigger the breakdown of endogenous H_2_O_2_ under acidic conditions of TME, but also neutralize excess GSH, giving them great potential for further integration with other therapies.

### Au-Based Materials

Because of the low-toxic, chemically stable and easy functionalization, Au-based nanomaterials, including nanospheres, nanorods, nanoshells, and nanocages, have been widely explored for biomedical applications [23–29]. Due to the excellent photoabsorption and thermal conductivity of Au, Au-based nanomaterials are mostly employed as photothermal agents [30]. Au itself cannot be utilized as PSs to generate ROS directly, but it can enhance PDT efficiency by triggering PSs to achieve high ^1^O_2_ quantum yield based on the luminescence resonance energy transfer (LRET) effect or by transferring energy to molecular oxygen for sensitizing the formation of ^1^O_2_ due to localized surface plasmon resonance (LSPR) [31]. In addition, LSPR of the gold nanorods, nanoshells and nanocage can be easily adjusted to the NIR region, enabling deeper tissue penetration and less photodamage [18]. As far as we known, few studies focused on Au-based H_2_O_2_-responsive materials for enhanced PDT have been reported till now.

**Fig. 4:**
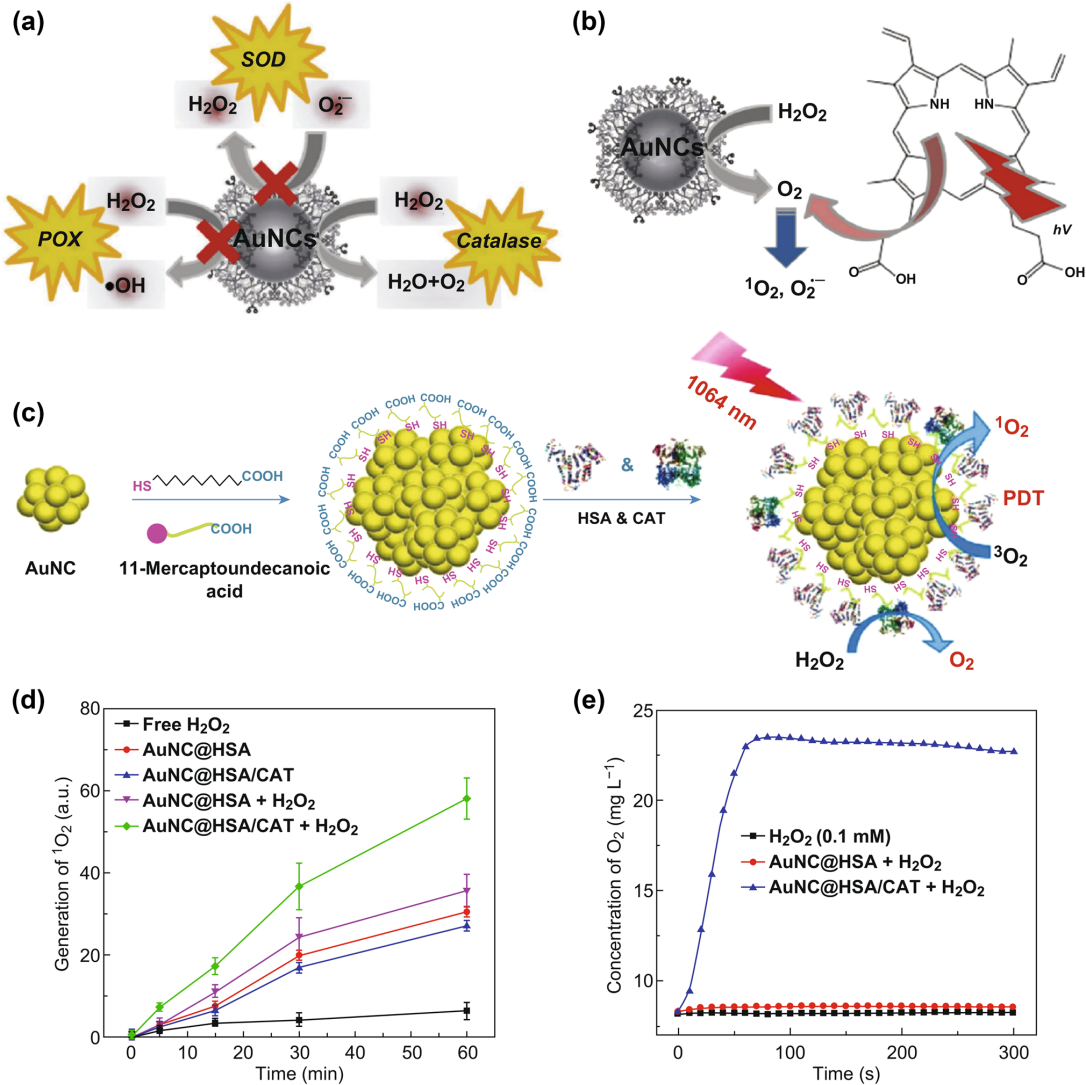
**a** Schematic diagram of enzyme-like activity of AuNCs. **b** Through the catalase activity of AuNCs-NH_2_, a simple strategy of combining traditional PDT with self-supplying O_2_ leads to increase in ^1^O_2_ and O_2_^•−^ generation. Adapted with permission from Ref. [32]. Copyright 2017 Wiley–VCH. **c** Schematic illustration of the synthesis of AuNC@HSA/CAT nanoparticles. **d** ^1^O_2_ generation for AuNC@HSA or AuNC@HSA/CAT with or without addition of H_2_O_2_. **e** O_2_ generation in H_2_O_2_ solutions (100 μM) incubated with AuNC@HSA or AuNC@HSA/CAT. Adapted with permission from Ref. [33]. Copyright 2017 Springer

The latest research about Au-based materials for H_2_O_2_-responsive phototherapy is reported by Lin’s group [32]. They successfully obtained amine-terminated PAMAM dendrimer encapsulated gold nanoclusters (AuNCs-NH_2_, Fig. 4a). The AuNCs-NH_2_ triggered oxygen production by reacting with H_2_O_2_ via the catalase-like activity in acidic TME. The proposed mechanism is that tertiary amines of dendrimers are easily protonated in acidic TME. The protonated products promote the pre-adsorption of ·OH on the metal surface, thus facilitating the intrinsic catalase-like reaction. With protoporphyrin IX (PpIX) in this platform, oxygen produced by AuNCs-NH_2_ via catalase-like reaction could be further converted into ^1^O_2_, thus enhancing the photodynamic therapeutic efficiency to some extent (Fig. 4b). However, due to the limited penetration of the visible light used to activate PSs, their strategy was only demonstrated in vitro rather than in vivo. This problem has been solved in another research by Liu and co-workers [33]. In their work, a gold nanorod-based NIR-II-responsive nanosystem was constructed, which was named as AuNC@HSA/CAT. Alkylthiolated gold nanoclusters (AuNCs) were co-modified with human serum albumin (HSA) and catalase (CAT) (Fig. 4c), which could be further used as a multifunctional nanoparticle. Under 1064 nm laser irradiation, cancer cell apoptosis could be achieved by chemically generated ^1^O_2_ and plasmonic heating from the gold nanorods with lower tissue absorption and scattering (Fig. 4d). Moreover, the presence of CAT in the nanoparticles triggered the decomposition of tumor endogenous H_2_O_2_ to generate oxygen, thereby enhancing the efficacy of PDT by relieving tumor hypoxia (Fig. 4e).

### Pt-Based Materials

**Fig. 5:**
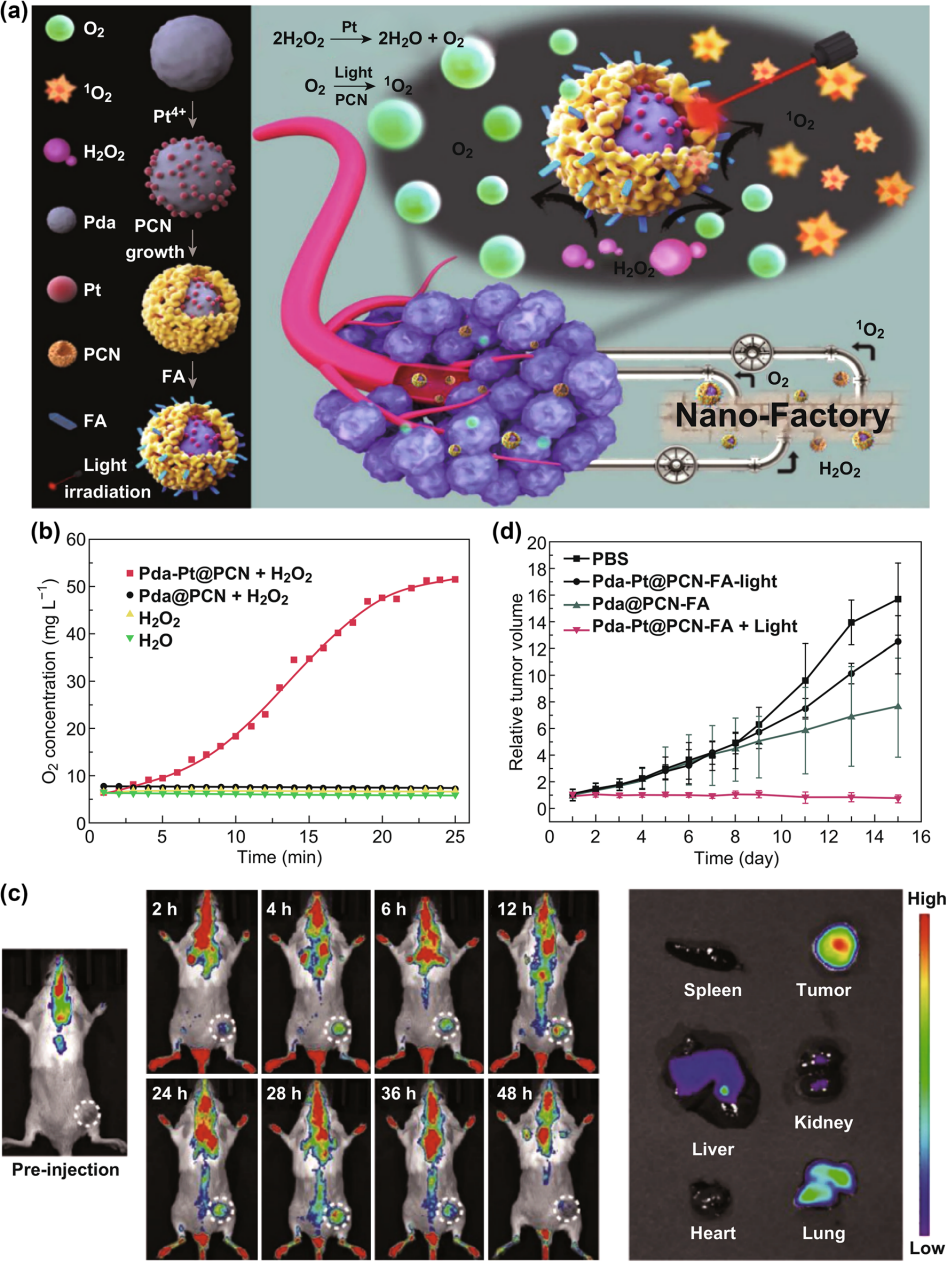
**a** Schematic illustration of core–shell nanofactory for enhanced tumor therapy. Pda-Pt@PCN-FA converts H_2_O_2_ from tumors into O_2_ and ^1^O_2_. **b** O_2_ generation of different groups. **c** In vivo fluorescence images of Pda-Pt @ PCN-FA at different time after intravenous injection. **d** Tumor growth volume curves of different treated mouse group. Adapted with permission from Ref. [35]. Copyright 2018 Wiley–VCH

Like Au-based nanomaterials, Pt-based nanomaterials can be easily surface-modified, combined with other drugs and demonstrate great ability to catalyze H_2_O_2_. Based on this, researchers try to develop several novel types of inorganic materials based on platinum for tumor PDT. One work worth mentioning is reported by Zheng’s group [34]. The authors chose Pd@Pt nanosheets as the substrate to covalently link with PS Ce6. After modification with PEG, the obtained Pd@Pt-PEG-Ce6 nanoplatform showed catalase-like activity, thus decomposing H_2_O_2_ into oxygen. Meanwhile, mild photothermal effects induced by Pd@Pt-PEG-Ce6 could increase intratumoral blood flow and enhance cellular uptake of PSs, leading to in situ O_2_ supplementation. Remarkably enhanced photodynamic therapy and photothermotherapy (PDT and PTT) were achieved both in vitro and in vivo owing to the relieved tumor hypoxia. In another work reported by Zhang’s group [35], Pt-based core–shell nanoplatform (Pda-Pt@PCN-FA) was developed for enhanced PDT via oxygen generation (Fig. 5a). Dopamine core was mixed with H_2_PtCl_6_, which was employed as Pt-based interlayer named Pda-Pt. Then, it was incorporated in zirconium–porphyrin (PCN) shell and immobilized with targeting ligands folic acid (FA) for enhanced tumor accumulation and desirable therapeutic efficacy. The Pt-based interlayer in this nanosystem could decompose endogenous H_2_O_2_ into O_2_, just like an oxygen-generation nanofactory (Fig. 5b). Under light irradiation, PCN shells converted the produced O_2_ into ROS, resulting in enhanced therapeutic efficacy of PDT. Meanwhile, O_2_ produced by H_2_O_2_ catalysis in dark will ameliorate the hypoxia, alleviating tumor invasion and metastasis. In vitro and in vivo studies have shown that this system could treat tumors more effectively by synergistically enhancing PDT and TME regulation (Fig. 5c, d). These studies not only enrich applications of Pt-based nanomaterials in cancer therapy, but also provide guidance for designing other nanosystems for cancer therapy.

### Fe-Based Materials

In recent years, Fe-based materials have received much attention in cancer treatment due to their high relaxation, excellent contrast enhancement, and biocompatibility. As a typical class of Fe-based materials, Fe_2_O_3_/Fe_3_O_4_-based materials have been used as magnetic resonance imaging (MRI) contrast agent and applied for magnetic hyperthermia in cancer treatment. In addition, Fenton reaction between Fe(II)/Fe(III) and H_2_O_2_ have been proved to be a direct ROS generation platform without light triggering [36]. Although Fe-based materials have been extensively studied for the detection of H_2_O_2_, their applications in PDT via H_2_O_2_ response have just appeared in recent years.

**Fig. 6:**
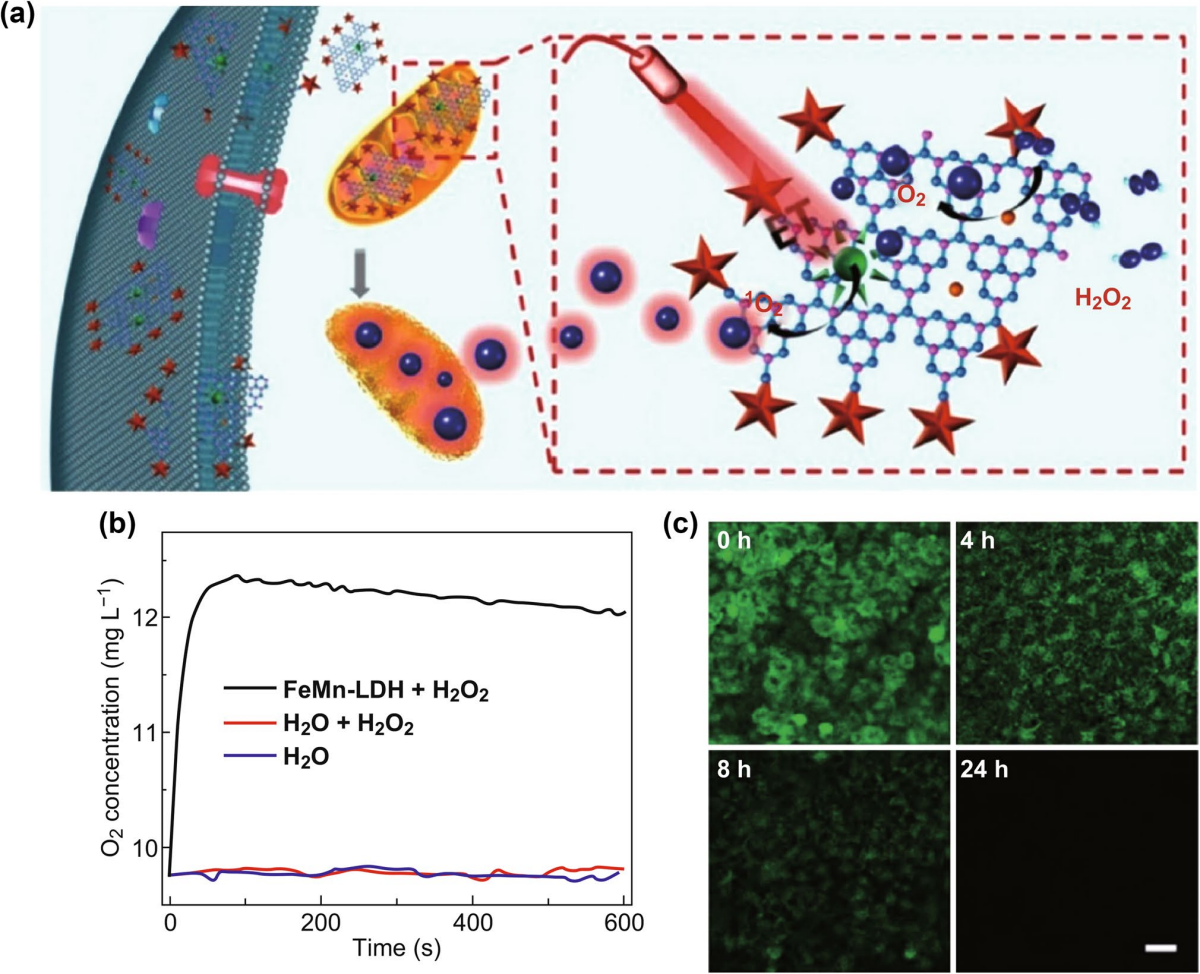
**a** Schematic illustration of C_3_N_4_-Fe-TPP NF/MB as theranostic nanocomposites for mitochondria-targeting H_2_O_2_-enhanced PDT. Adapted with permission from Ref. [37]. Copyright 2016 Wiley–VCH. **b** O_2_ was catalyzed by FeMn-LDH adding H_2_O_2_ (100 mM). **c** Evolution of O_2_ in HeLa cells incubated with O_2_ probe RDPP and FeMn-LDH (100 mg mL^−1^) at different time (0, 4, 8, and 24 h). Adapted with permission from Ref. [38]. Copyright 2018 Royal Society of Chemistry

For example, Jiang’s group [37] developed Fe(III)-doped two-dimensional (2D) C_3_N_4_ nanosheets for MRI and antitumor PDT. As a new type of 2D semiconductor material, graphite C_3_N_4_ (g-C_3_N_4_) nanosheet can be regarded as N-substituted graphite because of the six nitrogen lone-pair electrons in its π-conjugated structure and can be used for complexing or doping metal ions for functionalization (Fig. 6a). In this paper, C_3_N_4_ was doped with Fe(III), which resulted in excellent peroxidase-like catalytic performance. By catalyzing H_2_O_2_ to produce oxygen at the tumor site, the tumor hypoxia can be overcame and the PDT efficiency can be improved. Furthermore, after loaded with PS methylene blue (MB) and conjugated with mitochondria-targeting moiety (4-carboxybutyl) triphenylphosphonium bromide (TPP), a mitochondria-targeting photodynamic agent had been formed, which could evolve O_2_ into ROS by catalytic decomposition of H_2_O_2_. In addition, the loaded MB led to excellent photocatalytic performance toward O_2_ to generate ^1^O_2_, improving PDT efficiency. This development significantly overcame hypoxia in tumor tissue and broke the hypoxia limitation for PDT. Moreover, the ultra-high surface area of 2D nanosheets enhanced the PS loading capacity and could be used for effective interaction with tumor tissue for enhanced PDT efficiency.

Recently, Mn–Fe-layered double hydroxide (MnFe-LDH) nanosheets, new 2D nanomaterials, have been developed for O_2_-evolving phototherapy [38]. In general, LDH can release metal ions in response to the acidic microenvironment within the tumor, revealing its high contrast for MRI. However, the light absorption properties and biological catalytic activity of LDH has been neglected. It has been revealed that MnFe-LDH exhibited a broad NIR absorption spectrum due to small polaron absorption of oxygen defects. Moreover, the MnFe-LDH aqueous dispersion not only exhibited a great photothermal effect, but also could generate a large amount of oxygen when H_2_O_2_ was added (Fig. 6b, c). Oxygen-sensitive fluorescence probes have been applied and confirmed that both Mn^3+/4+^ and Fe^3+^ present effective H_2_O_2_ catalytic properties in this 2D nanosheets. The catalase-like activity of MnFe-LDH nanosheets enabled the decomposition of endogenous H_2_O_2_ into O_2_; therefore, tumor hypoxia could be overcame and therapeutic efficacy of O_2_-dependent PDT could be enhanced under laser illumination.

Another work based on Fe-Fenton theory is reported by Lin’s group [39]. In this work, the authors constructed FePt-NP2 nanoplatform using iron oxide as nanocarriers to load cisplatin (IV) prodrug. Nanoparticles could be preferentially accumulated in the tumor site via magnetic field-mediated localization and monitored by MRI. Specifically, in TME, cisplatin can activate the expression of nicotinamide adenine dinucleotide phosphate (NADPH) oxidase (NOX), which can transport electrons so that the O_2_ molecule can accept donated electron to generate ·O_2_^−^. Then, SOD catalyzes ·O_2_^−^ to form H_2_O_2_ which can be reacted with iron ions to improve Fenton-like reaction, resulting in highly toxic ·OH for enhanced PDT. In physiologically neutral or weakly acidic tumor environment, the Fe-Fenton reaction efficiency is relatively low. Even under ideal pH conditions, the Fe^2+^ catalyzed Fenton reaction has a low reaction rate (~ 63 M^−1^ s^−1^), which leads to slow ROS generation [40]. Therefore, it is highly desirable to develop nanoformulations with high catalytic activity and specificity in a weakly acidic TME.

### Cu-Based Materials

**Fig. 7:**
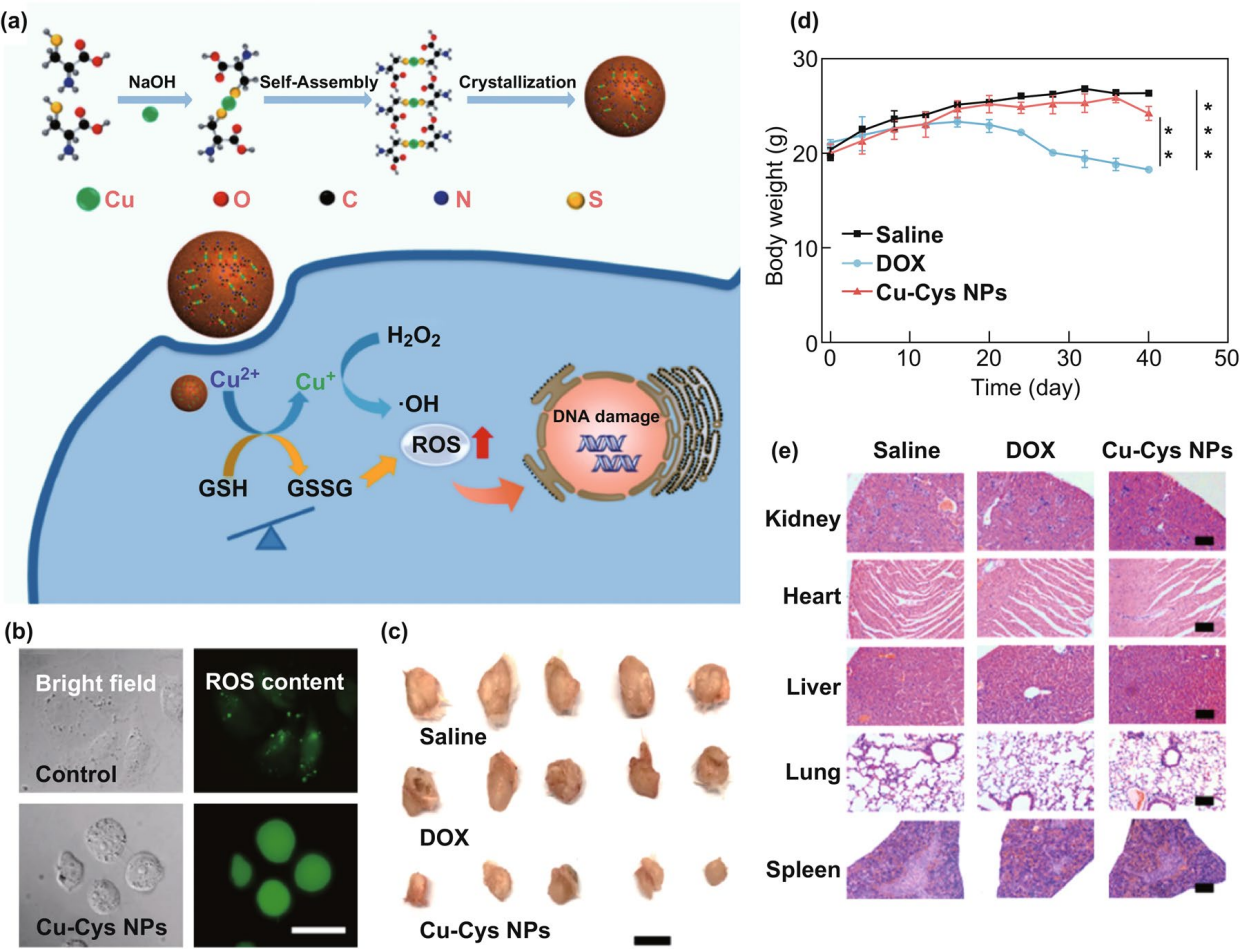
**a** Schematic diagram of the synthesis and action mechanism of Cu-Cys NPs. **b** Detection of ROS in ADSCs and MCF-7R cells before and after Cu-Cys NPs treatment. **c** Photographs of tumors collected from the mice. **d** Changes in body weight of mice during treatment. **e** Histological analysis of the major organs after treatment. Adapted with permission from Ref. [43]. Copyright 2018 American Chemical Society

Different from Fe^2+^-catalyzed Fenton reaction, the redox-active cuprous ion (Cu^+^)-catalyzed Fenton-like reaction is more advantageous in terms of kinetics and energy. It has been reported that a Cu^+^ catalyzed Fenton-like reaction can occur efficiently in weakly acidic and neutral media. Its highest reaction rate (1 × 10^4^ M^−1^ s^−1^) can reach ~ 160 times that of Fe^2+^ [41, 42]. Therefore, many works about Cu-based H_2_O_2_-responsive materials have been reported. Considering that copper ions have a high coordination ability with sulfhydryl group-containing ligands, Liu’s group produced novel copper-amino acid mercaptide nanoparticles named Cu-Cys NPs (Fig. 7a) [43]. Once the Cu-Cys NPs are endocytosed into cancer cells, GSH associates with the Cu-Cys NPs and reduces Cu^2+^ to Cu^+^. Then, Cu^+^ can react with H_2_O_2_ to generate Cu^2+^ and ·OH via the Fenton-like reaction, leading to DNA damage, protein inactivation, lipid peroxidation, and ultimately cell apoptosis (Fig. 7b). The in vivo experiment showed that tumor size could be effectively decreased after Cu-Cys NPs treatment, with a higher tumor suppressing potency in vivo (~ 72.3% inhibition rate) than the chemotherapeutic drug DOX (only 17.1% inhibition rate) (Fig. 7c, d). Besides, Cu-Cys NPs showed no significant damage on major organs, implying its great potential in cancer therapy with high specificity (Fig. 7e). Another work worth mentioning is reported by Jiang’s group. They prepared novel tetrakis(4-carboxyphenyl)porphyrin (TCPP)-modified 2D nanoscale metal–organic frameworks (nMOFs), Cu-TCPP, which can generate ^1^O_2_ by reacting with H_2_O_2_ and reduce the concentration of GSH in TME [44]. Cu-TCPP showed a novel ^1^O_2_ generation mechanism. The TCPP can be peroxidized by H_2_O_2_ at acidic pH and can be further reduced to peroxyl radicals (ROO·) and a trace amount of Cu^2+^ ions. Then, ^1^O_2_ can be produced in the spontaneous recombination reaction of ROO·. In addition, the consumption of GSH into oxidized glutathione (GSSG) by the incorporated Cu^2+^ ions which could prevent it from effectively scavenging ^1^O_2_ further increases the therapeutic efficiency. Different from previous strategies, the Russell mechanism has been utilized to produce ^1^O_2_ directly in the presence of trace metal ions. At the same time, biohydroperoxides can be produced directly by the peroxidation of ROS, which provides an alternative to oxygen-dependent photodynamic agents.

### Ce-Based Materials

As a rare-earth element, cerium has attracted much attention because of its strong redox ability and relatively high earth abundance. CeOx NPs have many advantages, such as low toxicity, catalytic activity, and adjustable absorption spectra. The interconversion between trivalent and tetravalent oxidation states plays an important role in biomedical and drug delivery. CeOx NPs show different enzyme mimics based on the ratio of Ce^3+^ to Ce^4+^. In addition, some studies indicated that CeOx NPs oxidase mimics could cause DNA damage, which made them cell killers at low pH. Therefore, Ce-based nanomaterials with the ability of catalase mimics and high tumor selectivity may be a more promising approach to achieve highly efficient PDT for future clinical treatment [45–47].

For example, Yang’s group developed a hollow-structured biophotocatalyst nanomaterials named UCNPs@mCeOx by coating mesoporous cerium oxide (mCeOx) on upconverting nanoparticles (UCNPs, NaGdF_4_: Yb, Tm@NaGdF_4_) [48]. The highly efficient and stable luminescent core–shell NPs can continuously convert 980 nm NIR laser into ultraviolet light, thereby exciting cerium oxide for PDT. The H_2_O_2_-responsive materials achieve sustained self-sufficiency of O_2_ during PDT, overcoming the obstacles of tumor hypoxia under near-infrared irradiation. In addition, inorganic UCNPs@mCeOx with high photostability is compared to conventional organic photosensitizers. Moreover, the huge hollow structure effectively loads the chemotherapeutic drug DOX, achieving synergistic PDT and chemotherapy.

**Fig. 8:**
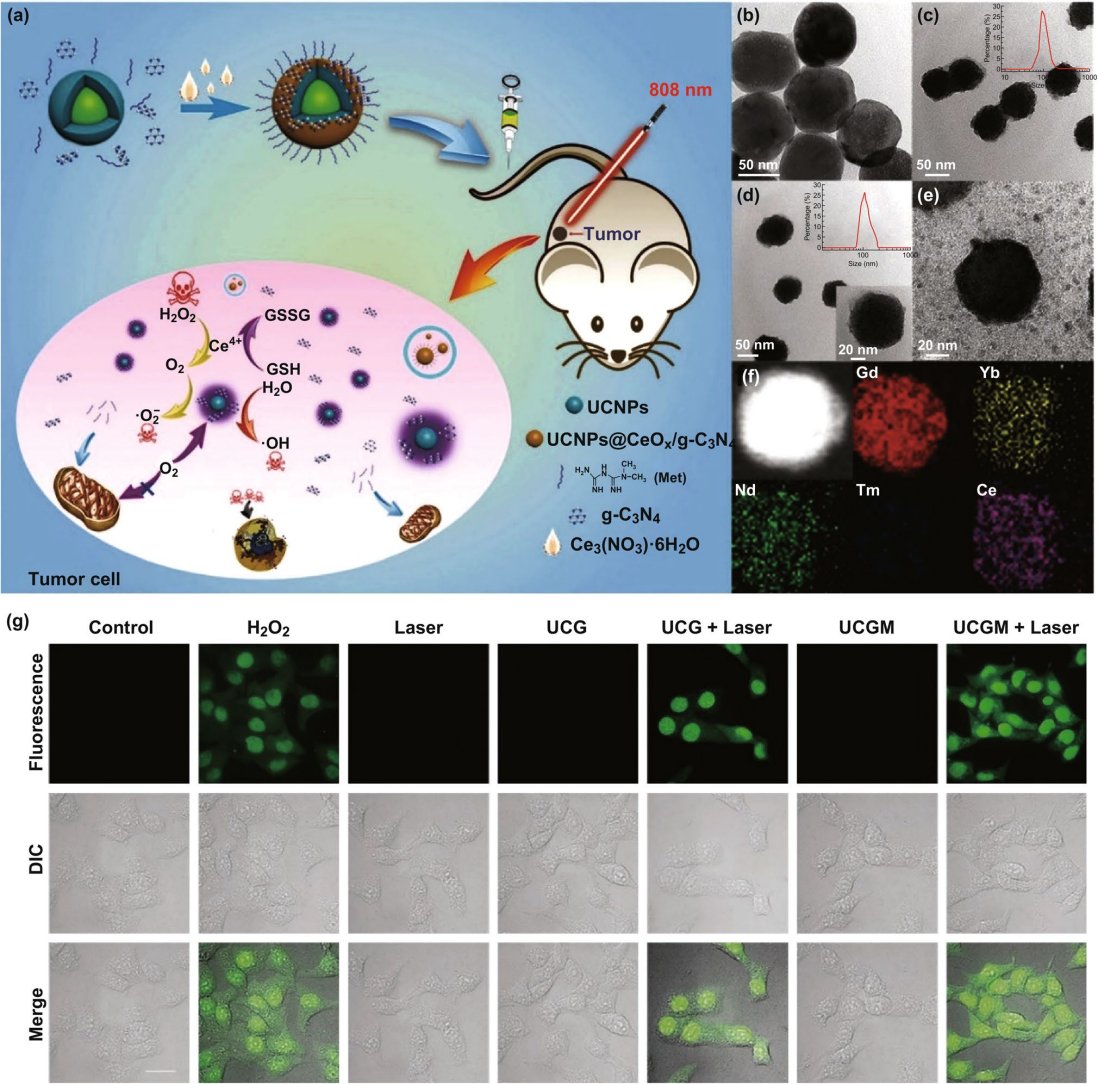
**a** Schematic diagram of the synthesis and action mechanism of UCNPs@CeOx/g-C_3_N_4_. **b** TEM image of NaGdF4:Yb, Tm@NaGdF_4_:Yb, Nd NPs. **c** TEM image of UCG NPs and the DLS curve of UCG NPs. **d** TEM images of UCGM NPs. **e** TEM images of UCGM NPs treated with H_2_O_2_ for 4 h under acidic condition (pH 6.5). **f** The EDS element mapping of UCGM NPs. **g** CLSM images of ROS generated in HepG2 cells with different treatment methods using H2DCFDA as indicator. Scale bar: 10 μm. Adapted with permission from Ref. [49]. Copyright 2019 Wiley–VCH

Ding’s group produced a multifunctional nanocluster bomb (UCGM nanoparticles) composed of UCNP NPs, CeOx, graphite-C_3_N_4_ (g-C_3_N_4_) NPs and metformin (Met) (Fig. 8a–f) [49]. CeOx could catalyze H_2_O_2_ into O_2_ to improve the oxygenation of TME. Meanwhile, Met can act on the mitochondria to inhibit the respiration of tumor cells to further increase O_2_ level. Under 808 nm laser irradiation, UCGM NPs have excellent photothermal capability, which could effectively convert near-infrared light into ultraviolet light, thus activating g-C_3_N_4_ NPs to produce ROS in tumor site and cause the tumor cell death. Further more, the UCGM NPs show excellent upconversion performance, magnetic resonance imaging and computed tomography imaging, making them potential imaging-guided drug delivery systems for cancer therapy (Fig. 8g). Great efficiency in combined PDT/PTT was achieved based on UCGM NPs.

In summary, a class of multifunctional nanomaterials with self-supplementing O_2_, NIR excitation, multimode imaging, and efficient ROS generation performance were synthesized for efficient PTT/PDT.

## Organic Materials for H_2_O_2_-Responsive Phototherapy

The common design for H_2_O_2_-responsive organic materials for enhanced cancer PDT is to immobilize organic PSs with H_2_O_2_-responsive moieties. H_2_O_2_-responsive moieties are functional groups with specific bonds such as boronic acid group [50], sulfide bond (–s–) [51], selenium bond (–Se–) [52], and tellurium bond (–Te–) [53]. Herein, we will provide an overview of current researches on H_2_O_2_-responsive drug delivery systems for enhanced PDT based on these functional groups.

### Aryl Boronic Ester-Based Materials

Boric acid is usually considered to be low toxic for humans and can be used in biomedical science. It is well-known that the esters of the aryl boronic acids can be cleaved by H_2_O_2_ and can be used in H_2_O_2_-activated fluorescent probes. Previous works have proved that phenyl boronic acid can be responsive to endogenous H_2_O_2_ to form a phenol in pathological conditions due to the oxidative stress caused by high level of H_2_O_2_ in cancer cells [54]. This reactivity provides a biologically compatible method for detecting endogenous H_2_O_2_.

**Fig. 9:**
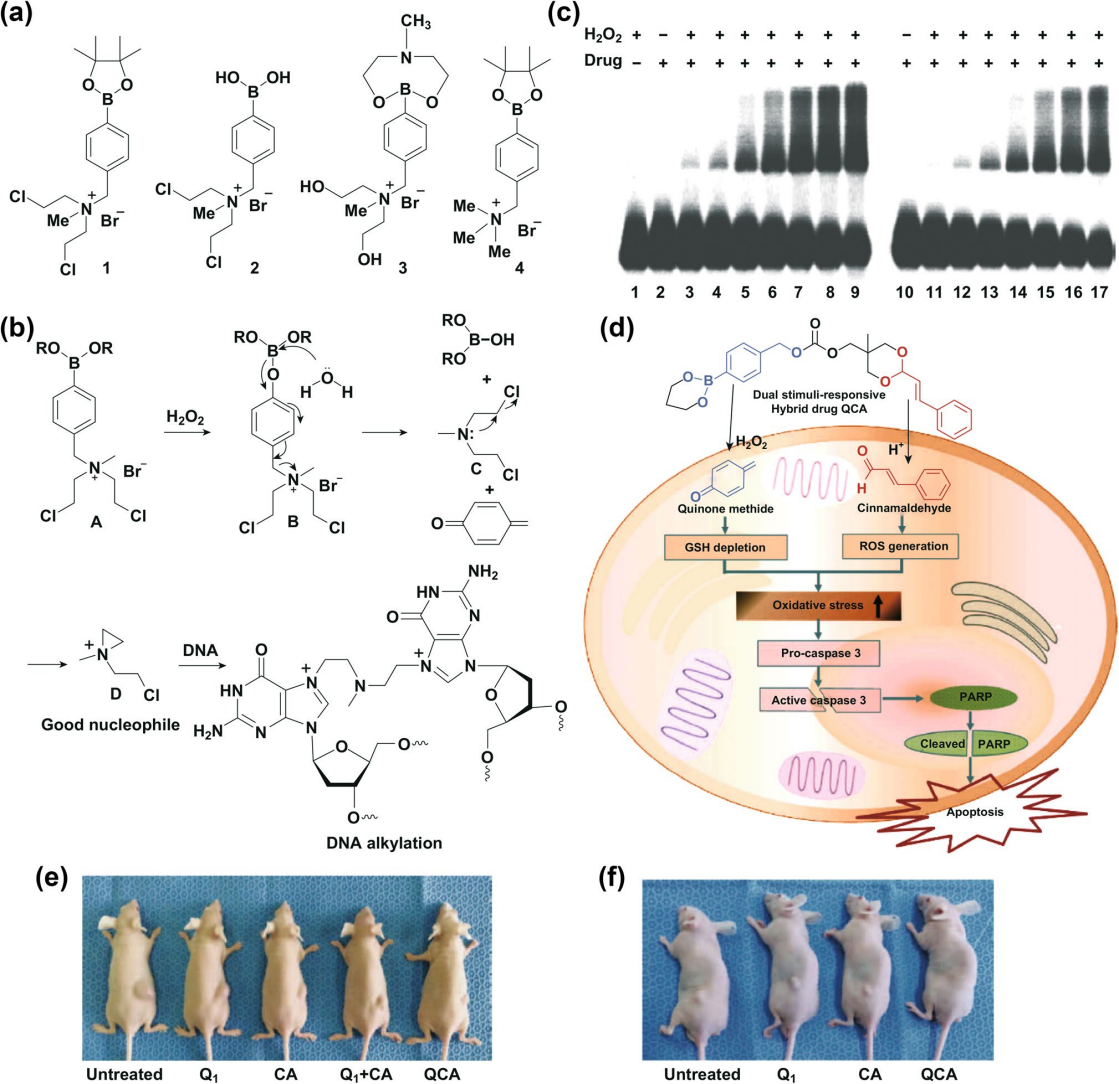
**a** Compounds 1–4. **b** Formation of interchain crosslinks (ICL) induced by 1 and 2 during H_2_O_2_ activation. **b** Release of nitrogen mustard from compound 1 upon treatment with H_2_O_2_. **c** The concentration dependence of compounds 1 and 2 on DNA cross-linking formation upon H_2_O_2_ activation. Lane 1 without drug; lanes 2–9 with drug 1: lane 2, without H_2_O_2_ (cross-linking yield 0%); lane 3, 50 μM H_2_O_2_ + 100 μM 1 (2.2%); lane 4, 100 μM H_2_O_2_ + 200 μM 1(5%); lane 5, 250 μM H_2_O_2_ + 500 μM 1 (11%); lane 6, 500 μM H_2_O_2_ +1.0 mM 1 (18%); lane 7, 1.0 mM H_2_O_2_ + 2.0 mM 1 (28%); lane 8, 1.5 mM H_2_O_2_ + 3.0 mM 1 (36%); lane 9, 2.0 mM H_2_O_2_ + 4.0 mM 1 (42%); lanes10–17 with drug 2: lane 10, without H_2_O_2_ (0%); lane 11, 50 μM H_2_O_2_ +100 μM 2 (2.0%); lane 12, 100 μM H_2_O_2_ + 200 μM 2 (4%); lane 13, 250 μM H_2_O_2_ + 500 μM 2 (11%); lane 14, 500 μM H_2_O_2_ + 1.0 mM 2 (17%); lane 15, 1.0 mM H_2_O_2_ + 2.0 mM 2 (27%); lane 16, 1.5 mM H_2_O_2_ +3.0 mM 2 (35%); lane 17, 2.0 mM H_2_O_2_ + 4.0 mM 2 (43%). Adapted with permission from Ref. [55]. Copyright 2011 American Chemical Society. **d** QCA is activated and degraded by H_2_O_2_ and acidic pH, leading to amplified oxidative stress and driving cells to undergo apoptotic death. **e** Representative images of mice bearing SW620 tumor xenografts. **f** Representative images of mice bearing DU145 tumor xenografts. Adapted with permission from Ref. [56]. Copyright 2015 Nature Publishing Group

Kuang and co-workers are pioneers in the design of nitrogen mustard prodrugs based on H_2_O_2_-activated aryl boronic ester [55], which could be activated by high level of H_2_O_2_ in cancer cells (Fig. 9a). These agents consist of two functional domains: the H_2_O_2_ acceptor moiety and the effector for cytotoxicity. A significantly increased cytotoxic potency can be achieved by releasing nitrogen mustard when triggering the aryl boronic ester with H_2_O_2_ (Fig. 9b). Their selectivity and antitumor activity is evaluated by comparing their effect on normal cells and cancer cells (Fig. 9c). It has been reported that both directly deliver ROS-producing agents to tumor tissues and inhibit the antioxidant system through destroying the redox balance in cancer cells which could remarkably enhance the ROS-mediated cancer cell killing efficiency. Based on that, Noh J.’s group synthesized [4-(1,3,2-dioxaborinan-2-yl)benzyl ((5-methyl-2-styryl-1,3-dioxan-5-yl)methyl) carbonate] (QCA) by coupling a quinone methide (QM)-generating moiety with ROS-generating cinnamaldehyde (Fig. 9d). Meanwhile, boronate was conjugated to cinnamaldehyde via a carbonate linker to achieve H_2_O_2_-responsive drug release [56]. The obtained product, QCA, can not only elevate intracellular ROS level through the reaction between cinnamaldehyde and endogenous H_2_O_2_, but also produce an antioxidant inhibitor quinone methide (QM) and triggering cell apoptosis. The in vitro and in vivo studies showed that cinnamaldehyde-mediated ROS production and the QM-induced oxidative stress elevation could result in enhanced apoptotic cell death (Fig. 9e, f). Herein, it is feasible to construct H_2_O_2_-responsive systems through a boron-based covalent bond to promote the intracellular generation of ROS and potentially to result in enhanced PDT. To the best of our knowledge, there is a growing trend referring to the research of H_2_O_2_-responsive materials based on aryl boronic groups. Aryl boronic acid and aryl boronic ester functional groups provide a promising way for enhanced PDT based on H_2_O_2_-response.

### Sixth Main Group Elements-Based Organic Materials

The sixth main group elements (oxygen group elements) mainly refer to oxygen (O), sulfur (S), selenium (Se), and tellurium (Te), which are all electronegative, easily losing their outermost electrons, and being oxidized by H_2_O_2_. In particular, the bond energies of C–Se, Se–Se, C–S, S–S, C–Te, and Te–Te are below 300 kJ mol^−1^, especially for S–S (264 kJ mol^−1^), Se–Se (193 kJ mol^−1^), and Te–Te (138 kJ mol^−1^), making the sixth main group elements-based agents more sensitive to H_2_O_2_ [57]. Sulfur is an essential element in organisms as well as an important component of protein in human body [58]. Selenium is the essential mineral nutrient in human body, which cannot be synthesized by ourselves but only rely on external supply. Researches show that selenium is very important for improving immunity and cancer prevention for its antioxidation property [59]. Below selenium is tellurium in the periodic table; Te-based agents are expected to be more sensitive to H_2_O_2_ than Se-based ones due to their higher electronegativity [60]. Meanwhile, tellurium-containing compounds have been used to develop new glutathione peroxidase (GPX) by imitating the natural antioxidant enzyme to protect cells from oxidative damage [61]. Moreover, studies show that organotellurium-containing compounds are less toxic than organoselenium-containing compounds [53]. These results inspire researchers to exploit the sixth main group elements-based agents for stimuli-responsive drug delivery systems. Herein, H_2_O_2_-responsive materials based on the sixth main group elements for enhanced PDT are discussed in detail (Table 1).

**Table 1:** H_2_O_2_-responsive bonds based on the sixth main group elements

Sixth group elements	Bonds
Oxygen	
Sulfur	
Selenium	
Tellurium	

#### Oxygen-Based Materials

Oxygen-based agents which are responsive to H_2_O_2_ typically contain functional groups such as aryl oxalate ester (AOE) and orthoesters. When exposed to very low concentration (~ 250 nM) of H_2_O_2_, AOEs are selectively cleaved at the oxalate group and undergo an autocatalytic mechanism, which would decompose into carbon dioxide rapidly. Orthoesters are compounds formed by the substitution of three hydroxy groups of alkoxy acids with alkoxy groups. Their general formula is RC (OR’)3, where R represents hydrogen or an alkyl group and R’ refers to an alkyl group. Orthoesters are extremely sensitive to mild acid and low concentrations of H_2_O_2_ and can be degraded by oxidation with H_2_O_2_.

**Fig. 10:**
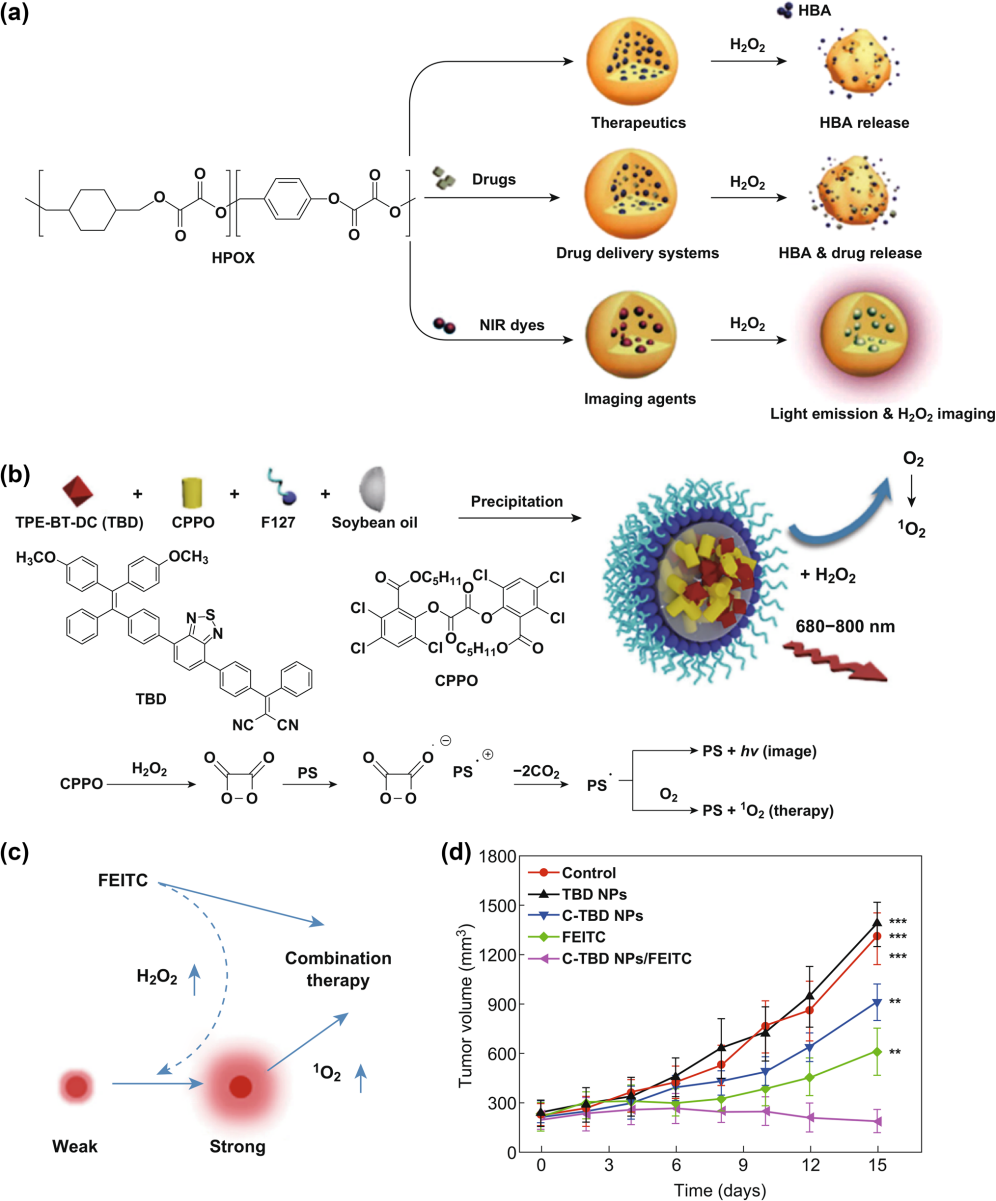
**a** Multifunctional H_2_O_2_-activatable nanoparticles for bioimaging and therapy. Adapted with permission from Ref. [62]. Copyright 2013 Elsevier Ltd. **b** The preparation of C-TBD NPs and illustration of the principle for chemiluminescence and ^1^O_2_ generation of C-TBD NPs in the presence of H_2_O_2_. **c** Schematic diagram of the hypothetical mechanism of C-TBD NPs and FEITC combination therapy. **d** Tumor growth curves with different therapies. Adapted with permission from Ref. [63]. Copyright 2017 Elsevier Ltd

Lee and co-workers successfully synthesized a biodegradable peroxalate copolymer named HPOX (Fig. 10a) [62]. This copolymer eligible to release active hydroxybenzyl alcohol (HBA) by a hydrolysis reaction in the presence of H_2_O_2_, accompanied by the formation of carbon dioxide (CO_2_). Meanwhile, the intramolecular oxalate structure would be degraded into oxalic acid under the same conditions. After this work, they also reported a series of HPOX-based systems for the diagnosis and remedy of oxidative stress-associated diseases. These special nanoparticles could be loaded with fluorescent dyes, PSs or drugs, demonstrating their potential for bioimaging, phototherapy and drug delivery.

Further research about H_2_O_2_-responsive systems based on AOE functional groups for enhanced PDT was reported by Lin’s group. A new C-TBD nanoparticle was formulated by co-encapsulation of bis[2,4,5-trichloro-6-(pentyloxycarbonyl)phenyl] oxalate (CPPO) and a specially designed AIE PS TPE-BT-DC (TBD) [63]. Considering that chemiluminescence is the luminescence typically produced by the release of energy through a chemical reaction between H_2_O_2_ and a high energy compound, it is a convenient technology with more clinical potential than bioluminescence and traditional fluorescence imaging since it does not require additional modifications. At the same time, the higher concentration of H_2_O_2_ in TME confers chemiluminescence good tumor selectivity. When CPPO-TBD is exposed to H_2_O_2_, the reaction between the aryl oxalate ester group in CPPO and H_2_O_2_ produces 1,2-dioxetanedione diketone. If the energy levels of the highest occupied molecular orbitals (HOMO) of TBD and the lowest unoccupied molecular orbitals (LUMO) of the intermediate are match, 1,2-dioxetanedione can directly stimulate TBD to produce ^1^O_2_ (Fig. 10b). In vivo experiments have shown that tumor imaging and therapeutic efficiency can be further improved when b-phenylethyl thiocyanate (FEITC, H_2_O_2_-enhanced agent) is introduced to increase the concentration of H_2_O_2_ in tumor (Fig. 10c, d). The novelty of this work is that it does not rely on external light sources, but far-infrared/near-infrared (FR/NIR) luminescence induced by aggregation can be used for accurate tumor imaging in vivo. These results prove that chemiluminescence imaging-guided PDT can be a promising noninvasive tool in cancer theranostics. Similar organic nanoparticles based on oxalate functional groups provide new ways to enhance PDT based on H_2_O_2_-responsive drug delivery systems.

#### Sulfur-Based Materials

According to the previous studies, compounds containing sulfide bond, especially thioethers or thiols, are usually used to react with H_2_O_2_. Thioethers are a group of compounds with the general formula R-S-R which undergo phase transition from hydrophobic to hydrophilic under ROS conditions, and thioether-based compounds have attracted much attention in stimuli-responsive drug delivery systems because thioethers could be oxidized into sulfones when exposed to H_2_O_2_. In addition, thiols are a class of nonaromatic organic sulfur compounds that contain thiol functional groups, which are usually applied as carbonyl protecting groups. In particular, thioketal bonds can be broken under oxidative condition. In recent years, large numbers of researchers started to focus on sulfide bond-based systems applied to H_2_O_2_-responsive drug delivery systems because of their unique oxidation properties. Based on this, the applications of H_2_O_2_-responsive drug delivery platforms based on sulfide bond in PDT have also been well reported.

**Fig. 11:**
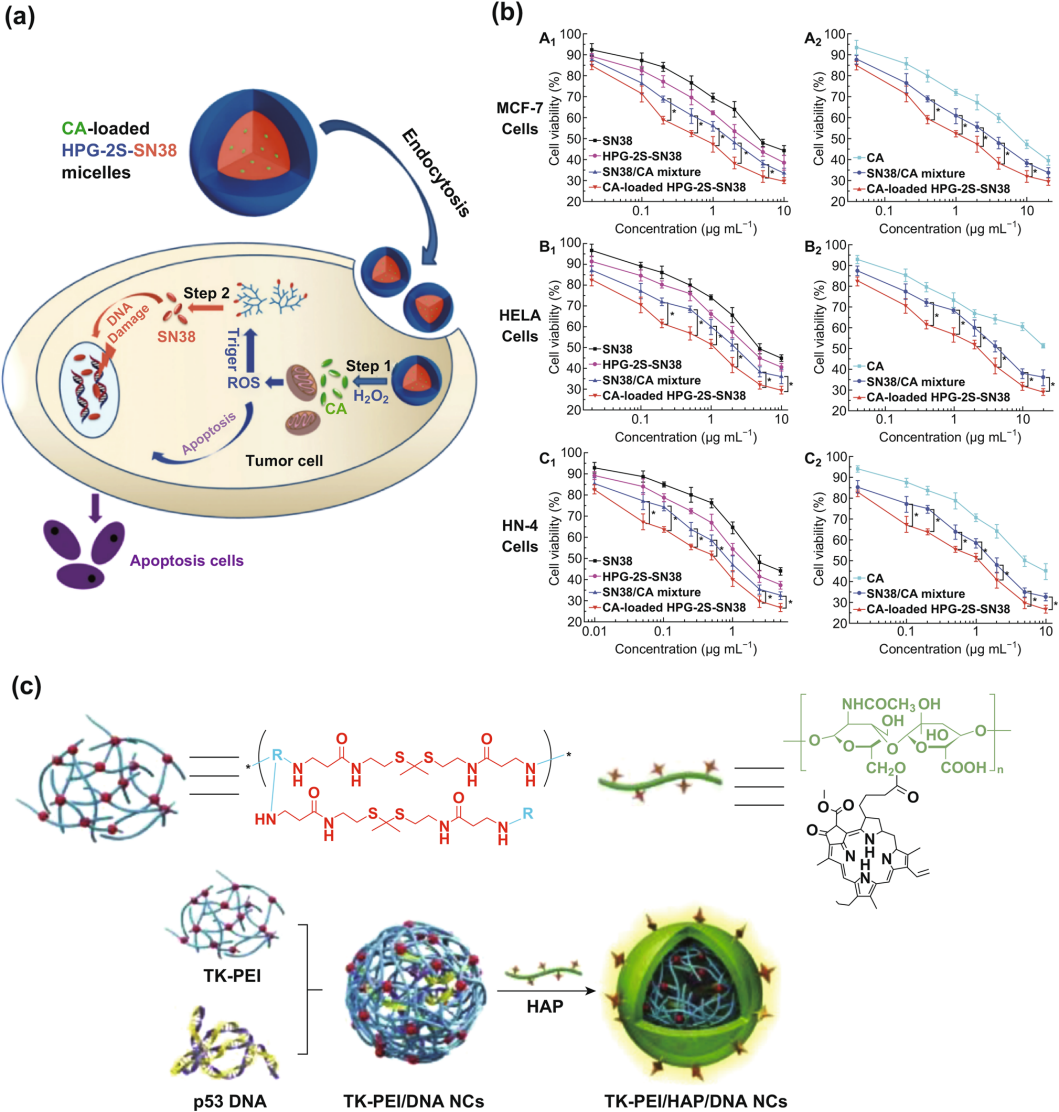
**a** Schematic diagram of the mechanism of apoptosis induced by CA-loaded HPG-2S-SN38 nanomicelles. **b** Cell viability was (A1, A2) MCF-7 cells, (B1, B2) HeLa cells and (C1, C2) HN-4 cells were treated with CA, SN38, HPG-2S-SN38 micelles after 48 h, physical mixture of SN38 CA and CA-loaded HPG-2S-SN38 micelles were at various SN38 (A1, B1, C1) and CA (A2, B2, C2) concentrations. Adapted with permission from Ref. [64]. Copyright 2015 The Royal Society of Chemistry. **c** Schematic illustration of light-assisted p53 gene delivery and PDT for cooperative anticancer therapy. Adapted with permission from Ref. [65]. Copyright 2018 Elsevier Ltd

Liu and co-workers used thioether as a linker to form the novel polymer micelles HPG-2S-SN38 [64], which was synthesized by conjugating hydrophilic hyperbranched polyglycerol (HPG) with 7-ethyl-10-hydroxycamptothecin (SN38), a hydrophobic antitumor drug. Cinnamaldehyde (CA) was then encapsulated into micelles to induce ROS production (Fig. 11a). Three typical cancer cell lines, MCF-7, Hela, and HN-4 were used to study the anticancer effect of HPG-2S-SN38. After entering into cancer cells, the HPG-2S-SN38 micelles were oxidized by oxidation of thioethers in the presence of H_2_O_2_, resulting in rapid hydrolysis of the phenolic esters in the structure of micelles, thus releasing CA and SN38 rapidly, resulting in an H_2_O_2_ concentration correlated synergistic antitumor effect. The evaluation of the cytotoxicity of HPG-2S-SN38 normal cells (L929 cells) ensures that H_2_O_2_ is the main pathway that triggers the oxidation of disulfide bonds and degrades HPG-2S-SN38, finally leading to cell apoptosis (Fig. 11b). This study demonstrates that H_2_O_2_-responsive polymer micelles constructed by sulfur-based bonds can enhance cell apoptosis through ROS production. For further improvement, it must be mentioned here that Yin’s group applied thioketal to construct a gene delivery system combined with PDT for tumor therapy [65]. ROS-degradable nanocomplexes (TK-PEI/DNA NCs) were successfully designed through thioketal-crosslinked polyethyleneimine (TK-PEI). After being coated with hyaluronic acid (HA), which was modified with pheophytin a (Pha) and co-delivered with a PS and p53 gene, TK-PEI/DNA NCs had colloidal stability (Fig. 11c). Upon intratumoral administration, NCs entered cancer cells via HA-assisted CD44 targeting, and then short-time visible light irradiation (661 nm, 5 m Wcm^−2^, 8 min) produced non-lethal levels of ROS to facilitate the endosomal escape of NCs via the photo-chemical internalization (PCI) effect, and simultaneously promote the intracellular DNA release by degrading the TK-PEI. After successful transfection to produce p53 protein, long-term visible light irradiation (661 nm, 5 m Wcm^−2^, 30 min) is generated for the process that internal light induces PSs to produce PDT effects. In this report, the size of TK-PEI/DNA NC changed significantly after H_2_O_2_ treatment, confirming that H_2_O_2_ induced cleavage of the thioketal crosslinker to degrade TK-PEI and dissociate TK-PEI/DNA NCs. It should be noted that among various ROS types, TK-PEI showed high sensitivity to superoxide and peroxide. These results clearly demonstrate that these block copolymer micelles are capable of drug-releasing when exposed to either oxidative (H_2_O_2_) or reductive (GSH) environment in vitro. At the same time, they can enhance PDT through multi-responsive release and multi-model collaboration.

#### Selenium-Based Materials

The Spector’s group first reported the antioxidant activity of selenium-based groups in 1989. In 1994, Iwaoka and Tomoda proposed a mechanism for the high sensitivity of diselenide bond to H_2_O_2_. When diselenoether was reacted with H_2_O_2_, the selenium ether rapidly catalyzed H_2_O_2_ into water and finally combined with hydroxide ions to form selenol [66]. Since then, a large number of researchers have put their efforts on the studies related to selenium-based groups due to their good activity in either oxidizing or reducing environment. When it comes to biological applications, selenium-containing compounds are well-known as antioxidants for its GPX activity. Meanwhile, selenium-based groups can be responsive to different external stimuli such as pH, light, radical, temperature, reductants and oxidants, thus having been widely used in stimuli-responsive drug delivery systems. Among these stimuli, H_2_O_2_-responsive drug delivery platforms based on selenium have been developed for enhancing tumor PDT recently.

**Fig. 12:**
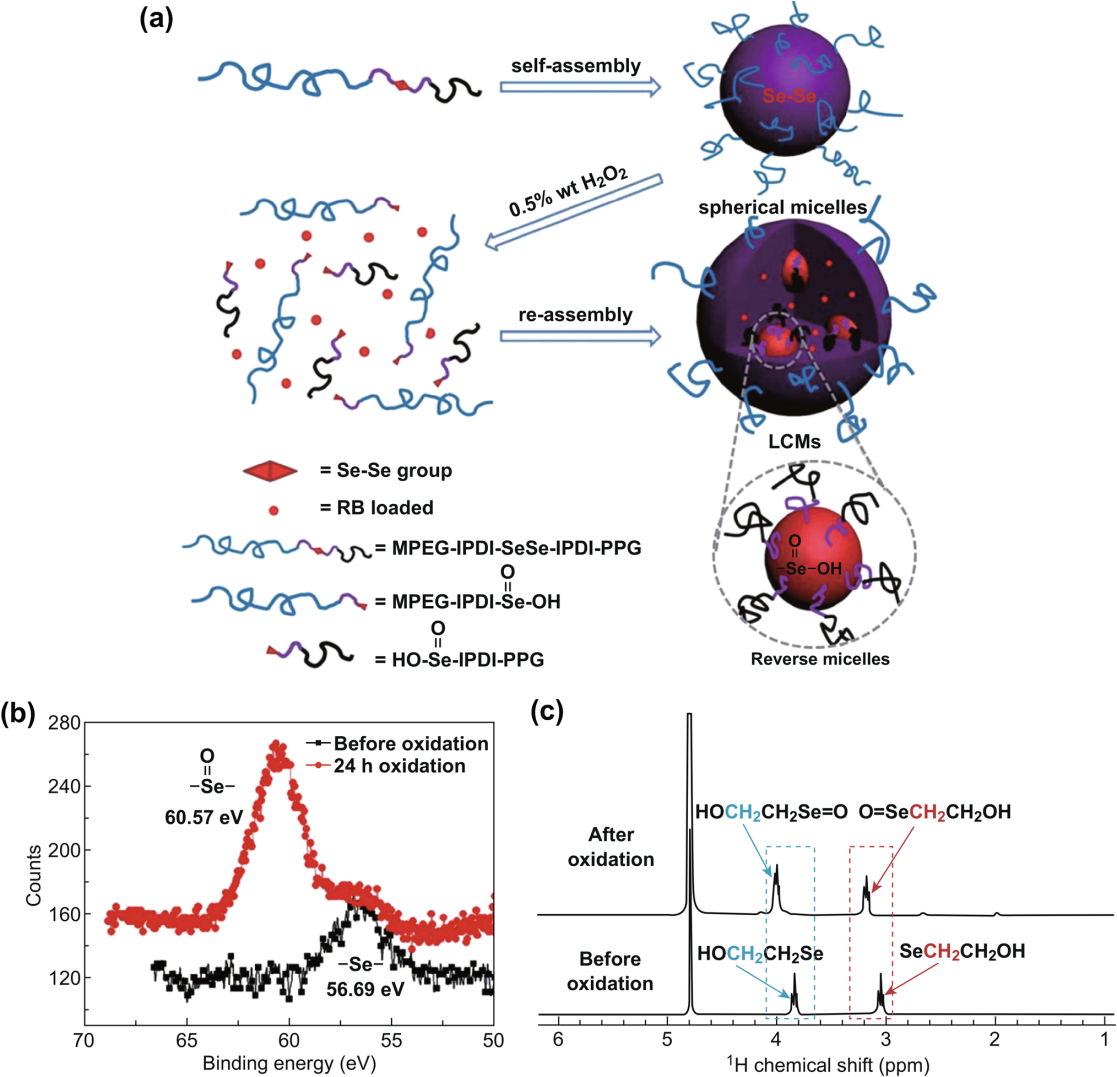
**a** Schematic of the oxidation-responsive morphological transformation of MPEG-IPDI-Se–Se-IPDI-PPG micelles. **b** XPS analysis of the model compound HOCH_2_CH_2_SeSeCH_2_CH_2_OH before and after oxidation in 0.5 wt% H_2_O_2_ solution. **c** ^1^H-NMR spectra of the model compound HOCH_2_CH_2_SeSeCH_2_CH_2_OH before (up) and after oxidation (down) in 0.5 wt% H_2_O_2_ solution. Adapted with permission from Ref. [67]. Copyright 2013 The Royal Society of Chemistry

Sun and co-workers have developed a variety of selenium-based polymers through orderly coupling reactions, such as MPEG–IPDI–Se–Se–IPDI–PPG (Fig. 12a) [67] and an amphiphilic triblock polymer called Se-Se-tri-ABP [68]. They are oxidation-responsive and can undergo morphological transformation when exposed to 0.5 wt% H_2_O_2_. The authors used these selenium-based polymers to load cargos, and the effective release of cargo in the H_2_O_2_ environment verified its great potential in stimuli-responsive drug delivery. Due to the similar properties and functions of these polymers, we selected one of the most representative researches for a detailed discussion. Amphiphilic block polymer (ABP) is the most promising drug delivery material due to its high physical loading capacity, adjustable size, and stable colloidal properties to prevent drug from inactivation. However, the drug release of ABP-based self-assembled micelles is slow and incomplete, which limits its application in cancer treatment. For enhanced therapeutic efficacy and reduced drug resistance, it is necessary to quickly release the cargo as completely as possible upon reaching the pathological site. To achieve this goal, a stimuli-responsive cleavage group, diselenide bond, is introduced into the MPEG–IPDI–Se–Se–IPDI–PPG. Due to the low bond energy of Se–Se, MPEG–IPDI–Se–Se–IPDI–PPG can be cleaved and oxidized to seleninic acid in the presence of oxidants and reduced to selenol in the presence of reductants. The change of the material in the system before and after the reaction of MPEG–IPDI–Se–Se–IPDI–PPG with 0.5 wt H_2_O_2_ was characterized by XPS analysis and ^1^H-NMR spectrum with HOCH_2_CH_2_SeSeCH_2_CH_2_OH as a reference. Confirm that H_2_O_2_ oxidizes the selenium bond to become selenite (Fig. 12b, c).

**Fig. 13:**
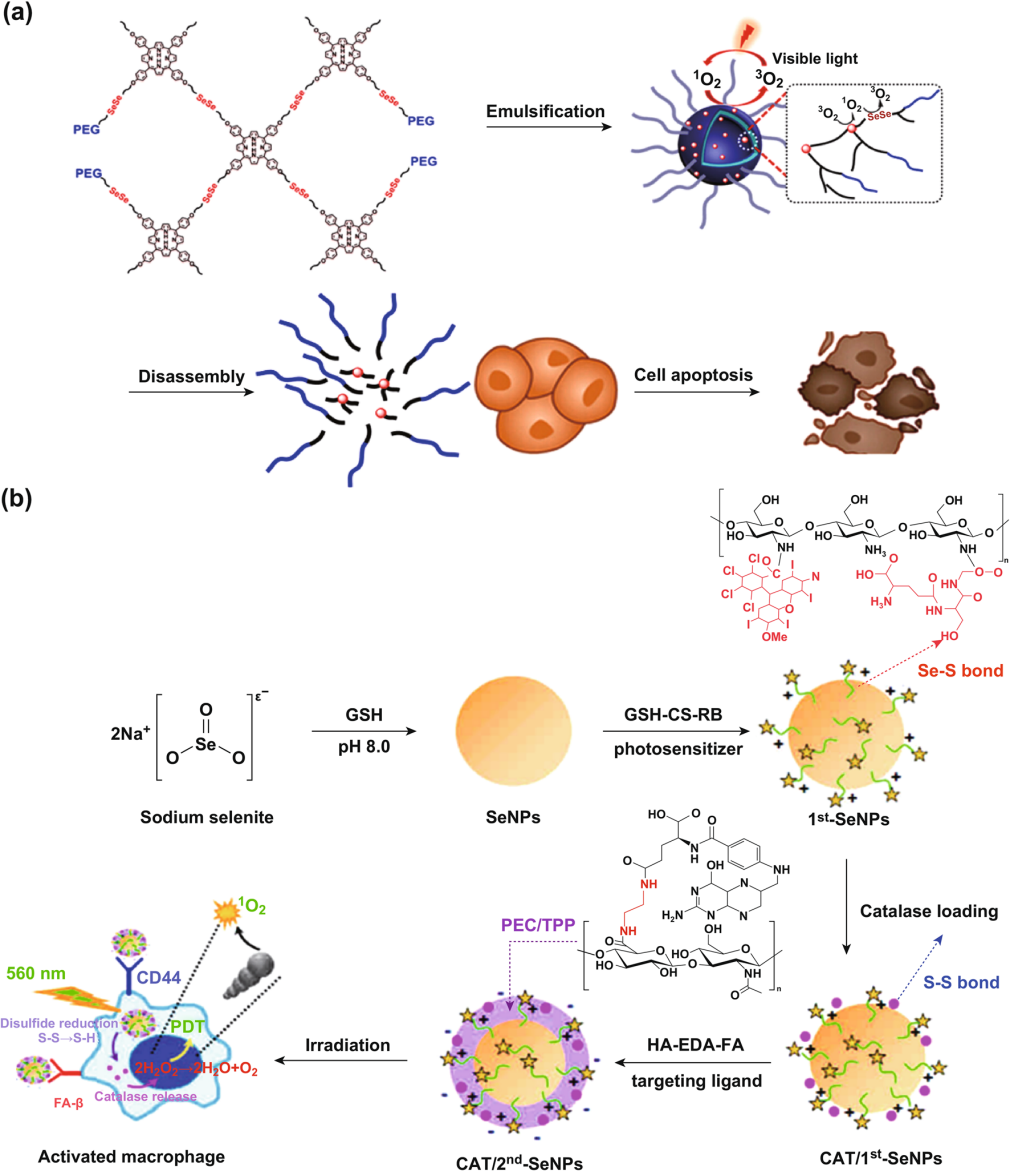
**a** Schematic illustration of the formation of the PSe-Por micelles. After light irradiation, the oxidization product has anticancer activity. Adapted with permission from Ref. [69]. Copyright 2017 American Chemical Society. **b** Schematic diagrams for the preparation of catalase-loaded SeNPs and photodynamic treatment. Adapted with permission from Ref. [70]. Copyright 2017 American Chemical Society

Moreover, Xu and co-workers successfully synthesized a series of H_2_O_2_-responsive block copolymers with selenium-based groups located at the polymer main chains or side chains which can be used for enhancing tumor PDT. One of the prominent researches is a supra-amphiphile diselenide-containing H_2_O_2_-responsive micelle (PSe-Por) with light-induced cytotoxicity [69]. Loaded with porphyrin derivatives, PSe-Por was sensitive to ^1^O_2_ when irradiated with visible light and could highly improve the use of singlet oxygen (Fig. 13a). Importantly, Mi’s group has constructed an H_2_O_2_-depleting and O_2_-generating platform based on selenium nanoparticles for fluorescence imaging and PDT [70]. In this study, photodynamic selenium nanoparticles (SeNPs) with photosensitive and macrophage-targeting bilayers were developed. Conjugated with a PS rose bengaL (RB) and a thiolated chitosan (chitosan–glutathione) via disulfide bonds in the first layer, the absorption intensity of photosensitive macromolecule increases and its absorption band has a great redshift induced by plasmonic coupling. Reduction in disulfide bonds through the intercellular decomposition of H_2_O_2_ can generate a large amount of O_2_ and then produce ^1^O_2_ under photoirradiation. Thus, the H_2_O_2_-depleting and O_2_-generating photodynamic SeNPs could efficiently kill activated macrophages, quenched the intracellular H_2_O_2_ and NO that are associated with inflammation and the SeNPs-based macromolecule may have potential as a theranostic nanomaterial in imaging and clinical applications (Fig. 13b). These open a new avenue for the development of H_2_O_2_-responsive drug delivery systems.

#### Tellurium-Based Materials

Tellurium-based materials with high selectivity and sensitivity toward H_2_O_2_ have been widely reported. As far as we know, most of them are inorganic materials. Similar to other organo-chalcogens, organotellurium-based compounds have been described as promising pharmacological agents due to their unique biological properties, especially for anticancer and antioxidant properties. Although plenty of evidence proves that they are presumed to have great potential for application in biological fields, there are few reports on the construction of the H_2_O_2_-responsive systems based on organotellurium to obtain desirable PDT efficacy.

**Fig. 14:**
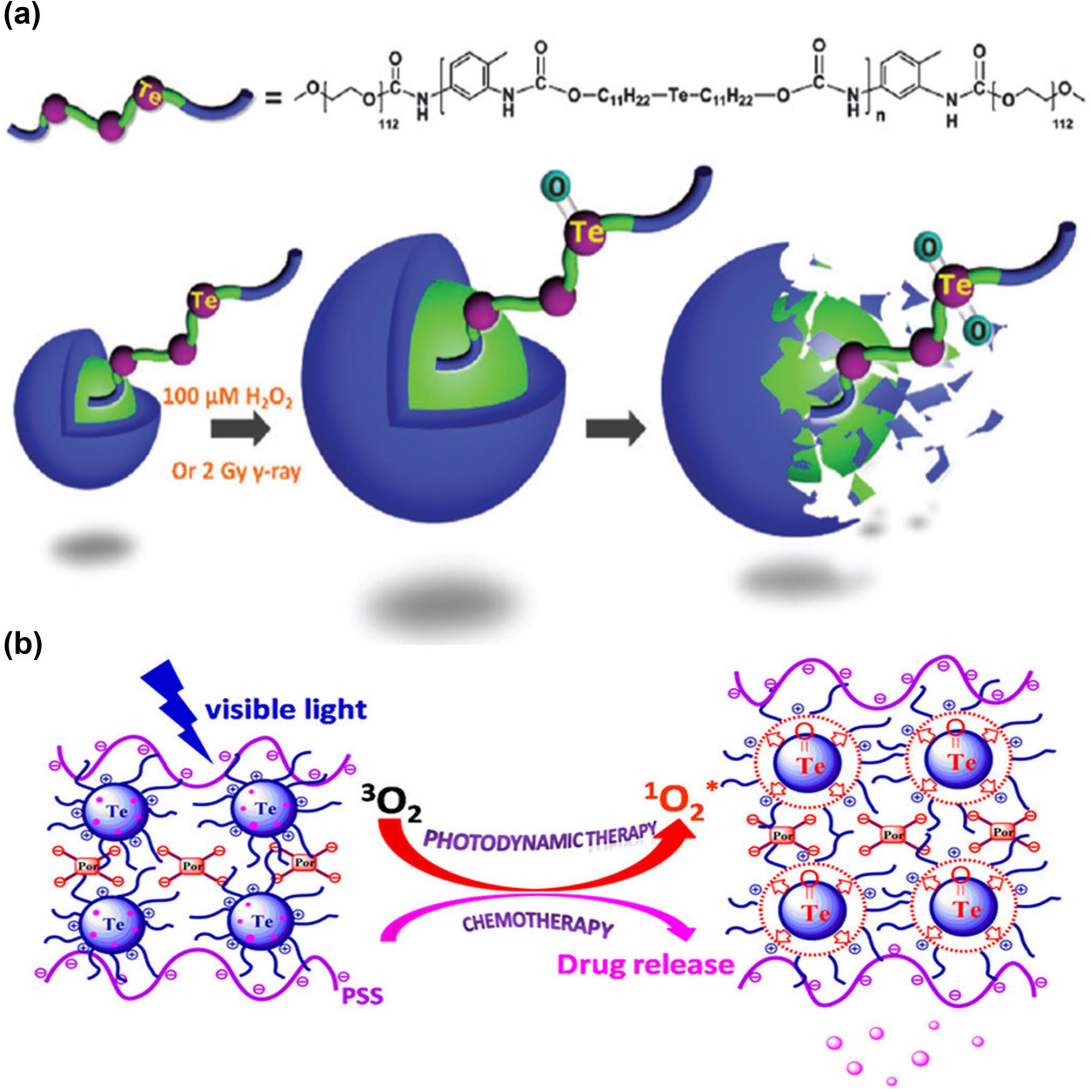
**a** Oxidation-responsive organotellurium-containing polymer micelles that could undergo a series of morphological changes triggered by ROS under biologically relevant conditions. Adapted with permission from Ref. [72]. Copyright 2014 American Chemical Society. **b** Visible light-responsive tellurium-containing multilayer film. Adapted with permission from Ref. [73]. Copyright 2016 American Chemical Society

Xu’s group has made a great contribution in this respect. Their first report about organotellurium-based stimuli-responsive polymer PEG–PUTe–PEG for controlled drug delivery systems was published in 2014 [71]. Competitive coordination of biomolecules can trigger the release of the loaded drug. This organotellurium-containing polymer, PEG–PUTe–PEG, swell and fracture dramatically when exposed to polyamines such as spermidine, arginine, and S-donor ligands such as biomolecules containing methionine and cysteine residues. This was confirmed by the variation of hydrodynamic diameters of the micelles. In order to confirm that the morphological transformation is caused by the oxidation of the telluride groups, ^1^H NMR spectra was used to show the different chemical shifts of the tellurium-containing segment. Based on these researches, in 2015 they successfully synthesized a series of H_2_O_2_-responsive block copolymers with selenium-based groups located at the polymer main chains or side chains which can be used as stimuli-responsive agents in drug delivery systems (Fig. 14a) [57, 72]. Moreover, they have also done some other studies based on organotellurium compounds for enhanced PDT [73]. Tellurium-containing photoresponsive polyelectrolyte multilayer films were fabricated by layer-by-layer assembly of a tellurium-containing polymer, PS, and poly(styrenesulfonate). Under visible light, the PS in the film is excited and transforms triplet oxygen into singlet oxygen in aqueous solution, which is important to enhance the efficacy of PDT. Singlet oxygen oxidizes (–Te–) to high valence state (Te–O) on the polymer backbone. The generated (Te=O) group makes the micelles more hydrophilic and looser, thereby facilitating the controlled release of the loaded cargo of micelles. These results indicate that the film has the potential for cargo loading and controlled release, providing a new approach to the combined PDT/chemotherapy (Fig. 14b). Above all, these organotellurium-based compounds provide an excellent platform for future biological applications.

## Summary and Outlook

In summary, H_2_O_2_ is an important endogenous ROS in human body and overproduced H_2_O_2_ is considered as a hallmark of malignancies. Importantly, H_2_O_2_ has also been studied as a stimulus in stimuli-responsive drug delivery systems. In this review, we have discussed the relationship between H_2_O_2_ and cancer briefly, and introduced the research progress of H_2_O_2_-responsive drug delivery system. More importantly, we summarized the emerging H_2_O_2_-responsive inorganic and organic materials for enhanced PDT. For inorganic materials, we have discussed the unique redox interaction between H_2_O_2_ and inorganic materials in detail, including multivalent metals Mn-, Au-, Pt-, Fe-, and Cu-based materials. Meanwhile, we emphasized their applications in enhanced PDT through distinct H_2_O_2_-responsive pathway. For organic-based materials, we discussed special functional bonds constructed from boron elements and sixth main group elements. The aryl boronic acids and their esters can be cleaved by H_2_O_2_ specifically, due to the strong oxidation performance of H_2_O_2_ and the electronegative property of sixth main group elements. With these achievements mentioned above, we firmly believe that these H_2_O_2_-responsive materials have great potential in enhancing anticancer PDT and they will play an important role in future cancer treatments. Further refinement of H_2_O_2_-responsive materials is also needed to make them more applicable to biomedical areas. At the same time, problems including tissue penetration depth, PS toxicity, tumor hypoxia, and short half-life of ^1^O_2_ remain to be further studied and resolved.
